# Using Incomplete Trios to Boost Confidence in Family Based Association Studies

**DOI:** 10.3389/fgene.2016.00034

**Published:** 2016-03-18

**Authors:** Varsha Dhankani, David L. Gibbs, Theo Knijnenburg, Roger Kramer, Joseph Vockley, John Niederhuber, Ilya Shmulevich, Brady Bernard

**Affiliations:** ^1^Institute for Systems BiologySeattle, WA, USA; ^2^Inova Translational Medicine InstituteFalls Church, VA, USA; ^3^School of Medicine, Virginia Commonwealth UniversityRichmond, VA, USA; ^4^School of Medicine, John Hopkins UniversityBaltimore, MD, USA

**Keywords:** family based association tests, missing genotypes, randomized imputation, quantile intervals, population stratification, whole genome analysis, memory efficient data format

## Abstract

Most currently available family based association tests are designed to account only for nuclear families with complete genotypes for parents as well as offspring. Due to the availability of increasingly less expensive generation of whole genome sequencing information, genetic studies are able to collect data for more families and from large family cohorts with the goal of improving statistical power. However, due to missing genotypes, many families are not included in the family based association tests, negating the benefits of large scale sequencing data. Here, we present the CIFBAT method to use incomplete families in Family Based Association Test (FBAT) to evaluate robustness against missing data. CIFBAT uses quantile intervals of the FBAT statistic by randomly choosing valid completions of incomplete family genotypes based on Mendelian inheritance rules. By considering all valid completions equally likely and computing quantile intervals over many randomized iterations, CIFBAT avoids assumption of a homogeneous population structure or any particular missingness pattern in the data. Using simulated data, we show that the quantile intervals computed by CIFBAT are useful in validating robustness of the FBAT statistic against missing data and in identifying genomic markers with higher precision. We also propose a novel set of candidate genomic markers for uterine related abnormalities from analysis of familial whole genome sequences, and provide validation for a previously established set of candidate markers for Type 1 diabetes. We have provided a software package that incorporates TDT, robustTDT, FBAT, and CIFBAT. The data format proposed for the software uses half the memory space that the standard FBAT format (PED) files use, making it efficient for large scale genome wide association studies.

## Introduction

A wide variety of genetic association studies have been performed with the aim of discovering genomic markers for a given phenotype of interest. While many of these studies are population based, there is a renewed interest in family-based studies due to the inability of population based studies to account for much of the heritability of most common phenotypes (Ott et al., 2011). Family based designs are commonly employed in genetic association studies because they are robust to population stratification (Laird et al., 2000). The two most widely used family based tests are Transmission Disequilibrium Test (TDT; Spielman et al., 1993) and Family Based Association Test (FBAT; Laird et al., 2000). TDT compares the frequency of allelic transmission from heterozygous parents to affected offspring. FBAT is a generalization of TDT that allows the use of families with unaffected offspring as controls, improving statistical power when studying common diseases (Lange and Laird, 2002). Both TDT and FBAT require complete genotypes. Any family where one or more members have missing genotypes is not used in these tests, resulting in loss of statistical power. Moreover, the genotypes are often not missing at random, but can be related to technical errors or to observed covariates. In such cases, ignoring or imputing missing genotypes can lead to systematic bias in the test statistic.

Several extensions to TDT have been proposed to handle missing data in affected families. Likelihood methods that deal with missing parental genotype information assume a homogeneous population in Hardy-Weinberg equilibrium (Van Steen et al., 2006). Croiseau et al. presented a multiple imputation approach for case-parent trio studies and showed it to have advantage of model flexibility over likelihood approaches (Croiseau et al., 2007). However, multiple imputation methods use posterior probabilities derived from the available data and as such, also assume a homogeneous population within the study cohort. Alternately, the robustTDT (Sebastiani et al., 2004) method handles incomplete genotypes without assuming any underlying patterns of missing data by exploring all possible genotype completions and returns upper and lower bounds of the TDT statistic (Sebastiani et al., 2004).

TDT-type tests are known to inflate the type I error rate where there is missing parental genotype information or undetected genotype errors or both (Van Steen et al., 2006). Moreover, most of the current genome wide association studies involve latent population substructure due to technical artifacts or diverse ancestries. Imputation methods that assume a homogeneous study population are not applicable in such cases. Cobat et al. proposed FBATdosage that computes the FBAT statistic by imputing missing genotypes using allele dosage (posterior mean genotype; Cobat et al., 2014). Here, we present a method to compute quantile intervals of the FBAT statistic (CIFBAT) without imputing missing genotypes. In this work we refer to the (α/2, 100-α/2) quantile intervals as “QIs,” where by default α = 0.05. These intervals are used to represent Z score and *p*-value spreads. CIFBAT computes QIs of the FBAT statistic by considering all valid completions of incomplete trios equally likely, and as such, does not assume homogeneous population allele frequencies. It includes families with unaffected offspring as controls, and most importantly, includes incomplete trios regardless of whether the parental or the offspring genotypes are missing. Table 1 compares various features between PLINK's implementation of TDT (Purcell et al., 2007; http://pngu.mgh.harvard.edu/~purcell/plink/), robustTDT (Sebastiani et al., 2004), FBAT (Laird et al., 2000), and our implementations of these methods, as well as CIFBAT. CIFBAT has been designed to analyze large numbers of whole genome sequences efficiently and can handle additive, dominant, and recessive genetic models for autosomal chromosomes, as well as the X chromosome. We have provided a software package called FamSuite with implementations of TDT, robustTDT, FBAT, and CIFBAT (https://github.com/IlyaLab/FamSuite). We present analysis of simulated genotype data to demonstrate applicability of CIFBAT in detecting bias in the FBAT statistic due to missing data and in identifying genomic markers with higher precision. We also present results from analysis of familial whole genome sequencing data set for maternal uterine anomalies and from a candidate marker data set for type 1 diabetes.

**Table 1:** **Comparison of features of TDT, robustTDT, FBAT, and CIFBAT**.

	**CIFBAT**	**FamSuite FBAT**	**FBAT (Laird et al., 2000)**	**FamSuite TDT**	**PLINK TDT**	**FamSuite robustTDT**	**robustTDT (Sebastiani et al., 2004)**
Unaffected offspring	✓	✓	✓				
Incomplete trios	✓					✓	✓
Support for ChrX	✓	✓	✓	✓	✓	✓	
Genetic models	A,D,R	A,D,R	A,D,R	A,D,R	A	A,D,R	A
Memory efficient data format	✓	✓		✓		✓	

*Original implementations of TDT, robustTDT and FBAT are compared with their implementations in FamSuite (For genetic models, A, additive; D, dominant; R, recessive)*.

## Materials and methods

In the following section, we refer to trios with disease affected offspring as “affected trios,” trios with unaffected offspring as “unaffected trios,” trios with one or more family members with missing genotypes as “incomplete trios,” and trios with at least one heterozygous parent as “informative trios.”

Since CIFBAT computes the FBAT statistic for every iteration of random completion of missing genotype data, we briefly recapitulate the FBAT parameters and statistic here. Further details about FBAT can be found in Laird et al. (2000).

### FBAT

The FBAT (Laird et al., 2000) statistic *Z*_*c*_ is based on the covariance *U*_*c*_ between the offspring's traits and genotypes:

(1) $$\begin{array}{ll} U_{c}=\sum_{i}T_{i}\left(X_{i}-E\left[X_{i}\right]\right)\; & \; \end{array}$$

(2) $$\begin{array}{ll} Variance\left(U_{c}\right)=\sum_{i}Variance\left(T_{i}X_{i}\right)\; & \; \end{array}$$

(3) $$\begin{array}{ll} T_{i}=Y_{i}-\;\mu \; & \; \end{array}$$

(4) $$\begin{array}{ll} Z_{c}=U_{c}∕\sqrt{Variance\;\left(U_{c}\right)}\; & \; \end{array}$$

Here, *X*_*i*_ denotes the offspring genotype in trio *i* at the genomic marker being tested. For a nuclear family with multiple offspring, there will be as many father-mother-offspring trios contributing to the test independently. The subscript “c” in the above formula denotes that FBAT is based on only “complete” trios in the data. *X*_*i*_ is defined by the genetic model (additive, dominant, recessive) under consideration. For example, for additive model, *X*_*i*_ counts the number of non-reference alleles observed in the offspring, and can take a value of 0, 1, or 2 for a bi-allelic genomic marker (Laird et al., 2000).

*T*_*i*_ is the coded trait defined as *Y*_*i*_ − μ, where *Y*_*i*_ denotes the observed trait of the offspring in trio *i*. Although Y can take several types of values, in this paper we focus on dichotomous traits where the observed trait *Y*_*i*_ is “1” for affected offspring and “0” for unaffected offspring.

μ∈**[0, 1]** is an offset value that can be chosen to maximize the power of the test (Laird et al., 2000). When μ = 0, *T*_*i*_ = *Y*_*i*,_ implying that only affected trios are used in the test (since *Y*_*i*_ is 0 for unaffected offspring). When μ > 0, affected trait *T*_*i*_ > 0 and unaffected trait *T*_*i*_ < 0, so both affected and unaffected trios are used in the test. For the analyses presented in this paper, we used μ = 0.5 in order to assign equal but opposite weights to affected and unaffected trios.

**Figure 1:**
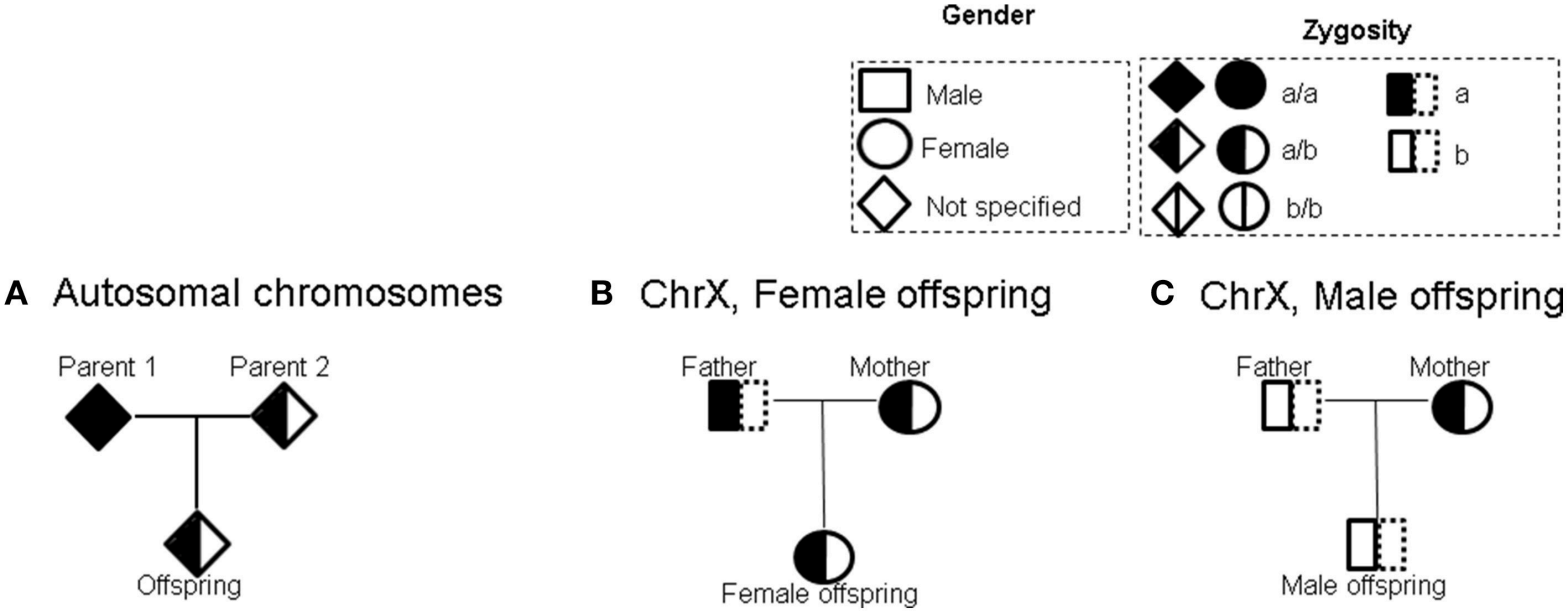
**Examples of informative complete trios. (A)** Autosomal chromosomes **(B)** X chromosome; trio with female offspring **(C)** X chromosome; trio with male offspring.

Figure 1A shows an example of an informative complete trio for autosomal chromosomes (Sebastiani et al., 2004). Figures 1B,C show examples of informative trio types with female and male offspring respectively for X chromosome. A comprehensive list of informative complete trios for autosomal chromosomes, as well as the X chromosome, is shown shown in Figure S1. The corresponding statistics *X* − *E[X]* and *Variance(X)* shown in Figure S1 are for the additive genetic model. Statistics for dominant and recessive models are in Table S1 (autosomal chromosomes) and Table S2 (X chromosome).

Here we describe an example to explain computation of the statistics *X* − *E[X]* and *Variance(X)*. For the trio type shown in Figure 1A where one parent is reference homozygous, the other parent is heterozygous and the offspring is heterozygous, under additive genetic model,

$$\begin{array}{l} X_{i}=1\\ E\left[X_{i}\right]=0*\;1∕2+1*\;1∕2=\;1∕2\\ X_{i}-E\left[X_{i}\right]=1∕2\\ Variance\left(X_{i}\right)=E\left[X_{i}^{2}\right]-{\left(E\left[X_{i}\right]\right)}^{2}\\ Variance\left(X_{i}\right)=\left(0*1∕2+1*1∕2\right)-{\left(1∕2\right)}^{2}\\ Variance\left(X_{i}\right)=1∕4 \end{array}$$

Trio types 6 and 7 for autosomal chromosomes (Figure S1) are not informative under the dominant model (Sebastiani et al., 2004) because both the possible offspring genotypes—heterozygous and alternate allele homozygous—have equal penetrance under dominant genetic model. Similarly, trio types 1 and 2 are not informative under the recessive model.

Once *X*_*i*_ − *E[X*_*i*_*], Variance(X*_*i*_*)*, and *T*_*i*_ are computed for each trio, *U*_*c*_ and *Variance(U*_*c*_*)* are computed by summation over all the trios and, finally, the FBAT statistic *Z*_*c*_ is computed as ratio of *U*_*c*_ and standard deviation of *U*_*c*_.

*Z*_*c*_ is essentially a z-score measuring deviation from the null hypothesis of no linkage and no association. When evaluating bi-allelic markers, a positive *Z*_*c*_ indicates that the allele being tested was over-transmitted to the affected offspring, whereas a negative *Z*_*c*_ indicates under-transmission to affected offspring. *P*-values are computed considering this as a two-sided test.

### CIFBAT—boosting confidence in FBAT with quantile intervals

The FBAT test described above does not account for incomplete trios in the study cohort. This might lead to undetected bias in the test statistic. We have implemented CIFBAT to detect potential bias in the FBAT statistic in presence of incomplete trios and to identify significant genomic markers with higher precision. CIFBAT considers all the valid completions of each incomplete trio equally likely and computes QIs of the FBAT statistic over many iterations of randomized completions.

**Figure 2:**
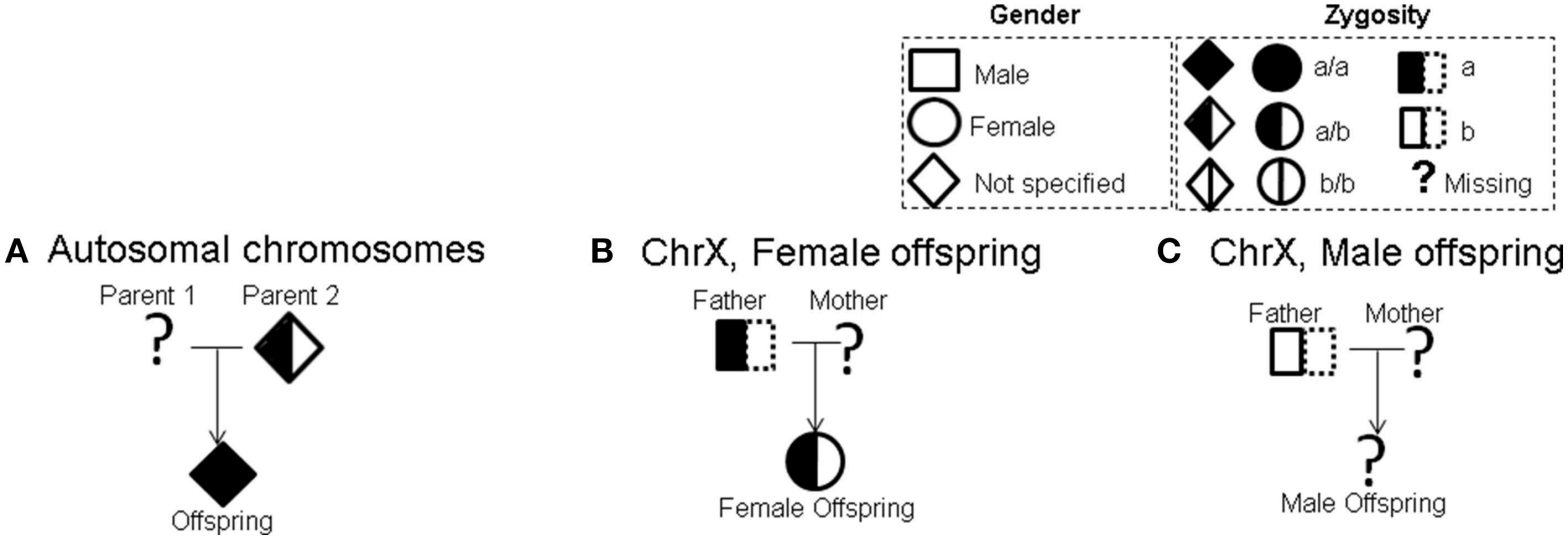
**Examples of admissible incomplete trios. (A)** Autosomal chromosomes **(B)** X chromosome (female offspring) **(C)** X chromosome (male offspring). CIFBAT considers all valid completions of incomplete trios in the data as equally likely. Using randomly selected completions over several repetitions, CIFBAT computes a quantile interval of the FBAT statistic.

Figure 2A shows an example of an admissible incomplete trio type for autosomal chromosomes (Sebastiani et al., 2004). Figures 2B,C show examples of admissible incomplete trio types with female and male offspring respectively for X chromosome. A complete list of all admissible incomplete trio types for autosomal chromosomes (Sebastiani et al., 2004) as well as X chromosome is shown in Figure S2. We computed *X* − *E[X]* and *Variance(X)* for all valid completions of these incomplete trios under additive, dominant, and recessive models respectively. Table S3 lists these statistics for autosomal chromosomes, and Tables S4, S5 list these statistics for the X chromosome for trios with male and female offspring respectively. For non-informative completions (both homozygous parents), both *X* − *E[X]* and *Variance(X)* are equal to 0.

We will now describe how CIFBAT computes QIs of the FBAT statistic. In the following explanation, subscript “*c”* denotes a complete trio or a statistic related to complete trios, subscript “*m”* denotes missing (incomplete) trio or a statistic related to incomplete trios, and subscript “*r*” denotes a random variable.

Suppose for a genomic marker under evaluation, our data set consists of *k* complete trios and d incomplete trios. The total *U*_*c*_ and Variance(*U*_*c*_) for all the complete trios are computed as:

(5) $$\begin{array}{l} U_{c}=\sum_{k}T_{k}\;\left(X_{k}-E\left[X_{k}\right]\right) \end{array}$$

(6) $$\begin{array}{l} Variance\;\left(U_{c}\right)=\sum_{k}T_{k}^{2}*Variance\;\left(X_{k}\right) \end{array}$$

Next, for each incomplete trio, CIFBAT chooses a completion assuming uniform distribution of all valid completions as listed in Tables S3–S5. Let *X'* denote the offspring's genotype for a randomly chosen completion. The total contribution of incomplete trios is a random variable *U*_*mr*_ computed as summation of contributions based on their random completions by CIFBAT:

(7) $$\begin{array}{ll} U_{mr}=\sum_{d}T_{d}\left(X_{d}^{{}^{\prime }}-E\left[X_{d}^{{}^{\prime }}\right]\right)\; & \; \end{array}$$

(8) $$\begin{array}{ll} Variance\left(U_{mr}\right)=\sum_{d}T_{d}^{2}*Variance\left(X_{d}^{{}^{\prime }}\right)\; & \; \end{array}$$

For a single iteration of random completion of all incomplete trios, the corresponding total *U* statistic and the variance are computed as the sum of the statistics from complete and incomplete trios.

(9) $$\begin{array}{ll} U_{total,r}=U_{c}+U_{mr}\; & \; \end{array}$$

(10) $$\begin{array}{ll} Variance\left(U_{total,r}\right)=Variance\left(U_{c}\right)+Variance\left(U_{mr}\right)\; & \; \end{array}$$

The corresponding *Z* statistic is computed as:

(11) $$\begin{array}{ll} Z_{r}=U_{total,r}∕\sqrt{Variance\left(U_{total,r}\right)} & \; \end{array}$$

For each genomic marker, CIFBAT executes a pre-defined number (1000 by default) of iterations of computing *U*_*mr*_ and the corresponding *Z*_*r*_, each time with a randomly selected set of completions for all incomplete trios. QIs of *Z*_*r*_ (α/2 and 100- α/2) and the corresponding *p*-values are then computed for a pre-defined confidence level (α = 0.05 by default).

### Simulation of family genotype data

In order to explore the statistical response of CIFBAT under various scenarios of missing data, we simulated family genotype data under no association and a genetic model of disease explained in detail in Data Sheet 1. The following three standard scenarios of missing data, as defined in the statistics literature, were simulated.

When data is “missing completely at random” (MCAR), the probability of an observation being missing does not depend on observed or unobserved measurements. This scenario was simulated by introducing missing genotypes for randomly chosen samples in the data. This type of missing data was use in the study of markers with no association.

Data can also be “missing at random” (MAR), which means that the missingness mechanism depends only on an observed measurement, and not on any unobserved measurements. This scenario was simulated with respect to the following observed variables separately: gender, subpopulation, and disease status.

Lastly, data can be “missing not at random” (MNAR) when the missingness mechanism depends on unobserved measurements. This can be difficult to detect and can lead to invalid inference if the missing data is ignored or incorrectly modeled. We simulated this scenario by concentrating missing data on heterozygous or homozygous samples, i.e., samples that were heterozygous or homozygous for the non-reference allele were more likely to be missing.

The simulated binary phenotype is either uniformly random or based on a bi-allelic additive genetic model closely following the work of Yang et al. (2003), where each individual starts with a low probability for disease and each causative SNP additively increases the probability. The model for log probability of disease, π_*ij*_, is defined as:

(12) $$\begin{array}{ll} \text{log}\left(\pi_{ij}\right)=\alpha_{ij}+\sum_{m}\beta_{m}X\left(G_{ijm}\right) & \; \end{array}$$

Here, α_*ij*_ is the initial log probability of disease for offspring *j* in family *i*. α represents environmental or other hidden factors that contribute to disease. For each causative SNP *m*, β is a log ratio of penetrance values comparing having one or two alleles to none. X(G) is a vector of dummy variables indicating a count of alternative alleles. Disease status was probabilistically determined using π_*ij*_; offspring within a family have correlated probabilities for disease, but are independent across families.

In each simulated scenario, 4000 pedigrees were generated and the mean population prevalence was 12.5%. An equal number of cases and controls were used, and missing data was generated at rates of 0, 1, 5, and 10% at the sample level (Table 2).

**Table 2:** **Simulation scenarios for comparison of FBAT and CIFBAT**.

**Missingness type**	**Missing data concentrated on:**
MCAR	Random
MAR	Small Pop.
MAR	Large Pop.
MAR	Males
MAR	Females
MNAR	Cases
MNAR	Controls
MNAR	Heterozygotes
MNAR	Homozygotes
Missing data was split by the above variables 80/20	

*Different missingness patterns (MCAR, MAR, MNAR) were simulated for comparing FBAT and CIFBAT. Population, gender, affectation status and zygosity were used to specify distribution of missing data for MAR and MNAR. In all, 9 scenarios were simulated at 0, 1, 5, 10% missing rate each*.

In the MAR scenario, for each level of missingness, we arbitrarily split the missing data 80/20 given a binary variable, such as gender or subpopulation, so that one population contains 80% of the missing data. The analysis was performed 1000 times for each level of missingness within each scenario, which results in 9 scenarios ^*^ 4 missingness levels ^*^ 1000 runs. The 9 scenarios are listed in Table 2. The parameters used and their range of values are listed in Table 3.

**Table 3:** **Parameters for simulation of family genotype data**.

**Parameter**	**Value**	**Notes**
Populations	2	Sized 1/3 and 2/3 of individuals
Families	4000	Each assigned to a population
Number affected offspring	636 (sd 294)	
Equal number of controls	Drawn from remainder of all pedigrees	
Number of offspring	Uniform random (1,2)	
Number of markers	300	
Number causative markers	3	
CIFBAT trials	100	
Penetrance with 2 causative SNPs	f_2_~ N(0.1, 0.01)	
Penetrance with 0 causative SNPs	f_0_~ N(0.001, 0.001)	
Environmental effect	λ_*s*_ = 3	
Genomic effect	λ_*g*_ = 2	
Marker Frequency	Gamma(shape = 2, scale = 2)/35.0	

A false discovery rate (FDR) cut-off of 10% was used to identify significant results. The equivalent *p*-value threshold (from the FDR) was also used to identify significant CIFBAT QIs. An interval was considered significant if the *p*-value spread was below the *p*-value threshold.

In each simulation, the causative SNPs are known. This allows computation of performance metrics useful for comparing CIFBAT and FBAT. Among the metrics computed are sensitivity (recall), the proportion of true positives called, specificity, the proportion of true negatives called, precision, proportion of predicted trues that are actually true, and the F-measure which is the harmonic mean of recall and precision.

### Real data: familial whole genome sequence data for maternal uterine anomalies

As more and more association studies employ whole genome sequences, it becomes important that novel association methods are evaluated for applicability and performance in genome wide association analyses. With this purpose, we used CIFBAT to analyze whole genome sequences of 784 nuclear families for uterine anomalies in mothers, represented as a binary phenotype. Samples of peripheral blood were collected from fathers, mothers and newborns at the Inova Fairfax Medical Center in Falls Church, Virginia, and sequencing was done at >40X depth using Complete Genomics' whole genome sequencing platform. Fifty-two (52) out of the 784 mothers were diagnosed with various uterine anomalies including endometriosis, bicornuate uterus, didelphic uterus, etc. (complete list in Text S1). These were used as cases in our study. Various filters were applied to the genomic markers to reduce noise in the data and to ensure applicability of family based association tests (Text S1). Altogether, we tested 3,808,482 autosomal markers under both FBAT as well as CIFBAT. A false discovery rate (FDR) cut-off of 10%, which translated to a *p*-value cut-off of 5.71e-05, was used to identify significant hits from FBAT. The *p*-value cut-off from FBAT results was also used to identify significant 95% QIs computed with CIFBAT. A QI was considered significant if the entire spread was below the *p*-value cut-off. A significant FBAT *p*-value together with a significant CIFBAT quantile interval indicated robustness against missing data. On the other hand, a significant FBAT statistic and a non-significant CIFBAT quantile interval hinted at potential bias in the FBAT statistic due to missing data.

### Real data: candidate marker set for type 1 diabetes

To demonstrate the use of CIFBAT in refining candidate sets of markers, we analyzed Type 1 diabetes data that consisted of a dichotomous phenotype variable and 351 markers within 22 candidate genes that have been previously identified as contributing to the risk of Type 1 diabetes. The data were collected by the Type 1 Diabetes Genetics Consortium (T1DGC). There were 2313 pedigrees consisting of 2345 nuclear families with one or more offspring. To ensure independence of nuclear families, we excluded one randomly selected family from every pair of families that had at least one common parent. We also excluded offspring whose type 1 diabetes affectation status was unknown as well as founders that had no sequenced offspring in the data. Finally, we were left with 2314 nuclear families with one or more offspring which we then analyzed using CIFBAT. An FDR cut-off of 10% was used to indicate significant results from FBAT. The equivalent *p*-value cut-off of 1.10e-02 was used to indicate significant results based on CIFBAT QIs. Again, a significant FBAT statistic with a significant CIFBAT quantile interval implied robustness against missing data, whereas a significant FBAT statistic with a non-significant CIFBAT quantile interval implied potential bias due to missing data.

### Extensions to TDT and robustTDT

In addition to developing the CIFBAT method, we have extended capabilities of the original TDT and robustTDT implementations as follows. PLINK (Purcell et al., 2007) is widely used for applying Transmission Disequilibrium Test (TDT) to family based studies. PLINK's implementation of TDT handles both autosomal chromosomes as well as the X chromosome, but only under an additive model. We extended our TDT implementation to handle dominant and recessive models in addition to the additive model. Table S6 lists the informative trios for autosomal chromosomes and transmission counts for the two alleles *b* and *c* under all three models. Tables S7, S8 list the informative trios with male and female offspring respectively for the X chromosome. Similar extensions were added to robustTDT (Sebastiani et al., 2004) to handle missing genotypes for the X chromosome in addition to autosomal chromosomes under all the three genetic models (Tables S9–S11).

## Results

### Simulated family genotype data

Analysis of simulated family genotype data allowed us to compare the performance of FBAT and CIFBAT under no phenotype association and the various patterns of missing data (MCAR, MAR, MNAR). The simulated scenarios of missing data are not meant to be comprehensive, but rather illustrative of the effects on algorithms that use missing data in making inferences. Comparisons were primarily based on recall and precision.

When phenotypes were uniformly random, and data was missing completely at random, the FBAT *p*-values remained uniformly distributed, resulting in 10% false positives at *p*-value threshold of 0.1 (with no FDR correction). CIFBAT Z_r_ score QIs, based on the interval median, were normally distributed around zero (see Figure S12). As the amount of missing data increases, the intervals widen, and fewer intervals lie completely below a given threshold. At 1% missing data 5.1% of *p*-value intervals were completely below 0.1 while at 10% missing data 0.3% of *p*-value intervals were below 0.1. However, when the CIFBAT method is used as described (computing FDR based on FBAT and comparing intervals to the *p*-value threshold), zero simulated markers are found to be significant at a 10% FDR level, as desired.

**Figure 3:**
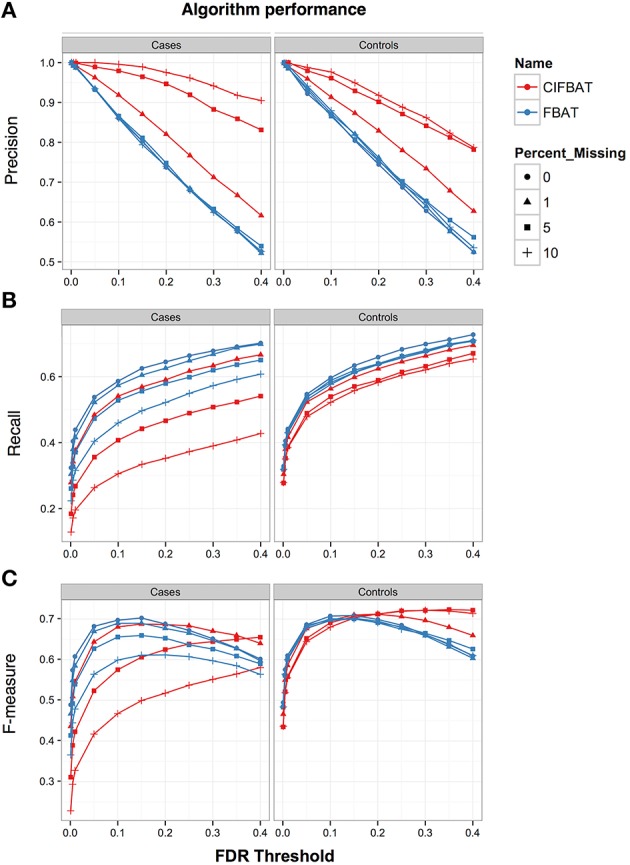
**Performance of FBAT and CIFBAT under Missing At Random (MAR) simulation scenario**. Shown is the **(A)** Precision, **(B)** Recall, and **(C)** F-measures related to calling the causative variant. Missing data is concentrated within cases or controls and performance is measured for different missing data rates and FDR thresholds.

Across all scenarios, for both FBAT and CIFBAT, the smallest effect on the recall occurred when missing data was concentrated on the controls, and the largest effect occurred when missing data was concentrated on the cases. In general, the cases will have more occurrences of a causative variant, so when missing data is concentrated on the cases, it weakens the power of the tests to detect the overabundance of causative alleles in cases, leading to false negative results. On the other hand, when missing data is concentrated on controls, it does not affect the signal to the same extent. Figure 3A shows the difference in recall for a sliding FDR threshold when missing data is concentrated on the cases or controls.

The simulations showed that overall, in comparison to FBAT, CIFBAT tended to trade lower recall for higher precision; meaning that while fewer variants were called significant, they were more likely to be true positives (Figure 3B). For example, in the scenario where missing data was concentrated in cases, the CIFBAT recall fell from 0.48 at 1% missing data, to 0.26 at 10% missing data, compared to 0.52 at 1% to 0.40 at 10% for FBAT (Table 4). However, the CIFBAT precision rose from 0.96 to 1.0 over the same range of missing data, compared to 0.93 for all ranges of missing data with FBAT (Table 4). CIFBAT specificity and negative predictive value remained above 99% over all missingness scenarios and levels (Table S12). This suggests that CIFBAT can be useful in detecting potential bias in the FBAT statistic due to missing data and in refining the set of significant markers to be validated for downstream analyses.

**Table 4:** **Results from analysis of simulated familial genotype data**.

**Algorithm**	**Mode**	**Missing rate (%)**	**Missing data concentrated in:**	**Recall**	**Precision**	**F-measure**
CIFBAT	MAR	0	Controls	0.547	0.922	0.686
	MAR	1	Controls	0.523	0.959	0.677
	MAR	5	Controls	0.490	0.976	0.653
	MAR	10	Controls	0.484	0.970	0.646
	MAR	0	Cases	0.538	0.933	0.682
	MAR	1	Cases	0.484	0.962	0.644
	MAR	5	Cases	0.356	0.989	0.524
	MAR	10	Cases	0.263	1.000	0.417
FBAT	MAR	0	Controls	0.547	0.922	0.686
	MAR	1	Controls	0.540	0.936	0.685
	MAR	5	Controls	0.537	0.931	0.681
	MAR	10	Controls	0.530	0.941	0.678
	MAR	0	Cases	0.538	0.933	0.682
	MAR	1	Cases	0.522	0.934	0.670
	MAR	5	Cases	0.473	0.932	0.627
	MAR	10	Cases	0.404	0.934	0.564

*Performance of FBAT and CIFBAT was compared based on recall, precision, and F-measure. CIFBAT tended to trade lower recall for higher precision; meaning that while fewer variants were called significant, they were more likely to be true positives. F-measure was comparable between FBAT and CIFBAT over all the simulation scenarios; however, the variance of F-measure was higher for CIFBAT*.

Ultimately, the levels of recall and precision are influenced by the disease model parameters. For example, changes in recall and precision can be seen in Figures S3, S4 respectively when the mean penetrance is varied in the model. A complete report of changes in various performance metrics of FBAT and CIFBAT with changes in model parameters is given in Table S12.

Overall, the F-measure, which is the harmonic mean of precision and recall, was comparable between FBAT and CIFBAT, with FBAT having a median F-measure of 0.67 and CIFBAT having a median F-measure of 0.64 over various simulation scenarios (Table 4). However, the variance of F-measure was greater for CIFBAT (Figure 3C and Figure S5).

The simulation also generated results where the CIFBAT QIs were significant but the FBAT statistic was not, although these were few in number (5/300) and no clear pattern emerged to explain their occurrence. Figures S6, S7 show how precision and recall of FBAT and CIFBAT are affected by percentage of missing data. While precision of CIFBAT is higher than FBAT in presence of missing data, its recall decreases with missing data. Figures S8, S9 show effect of the FDR threshold chosen to indicate significant results.

### Familial whole genome sequence data for maternal uterine anomalies

We analyzed familial whole genome sequencing data to identify potential genomic markers for maternal uterine anomalies. Further, we utilize this section to explain interpretation and usage of CIFBAT QIs in validating robustness of FBAT results in presence of missing genotype data.

**Figure 4:**
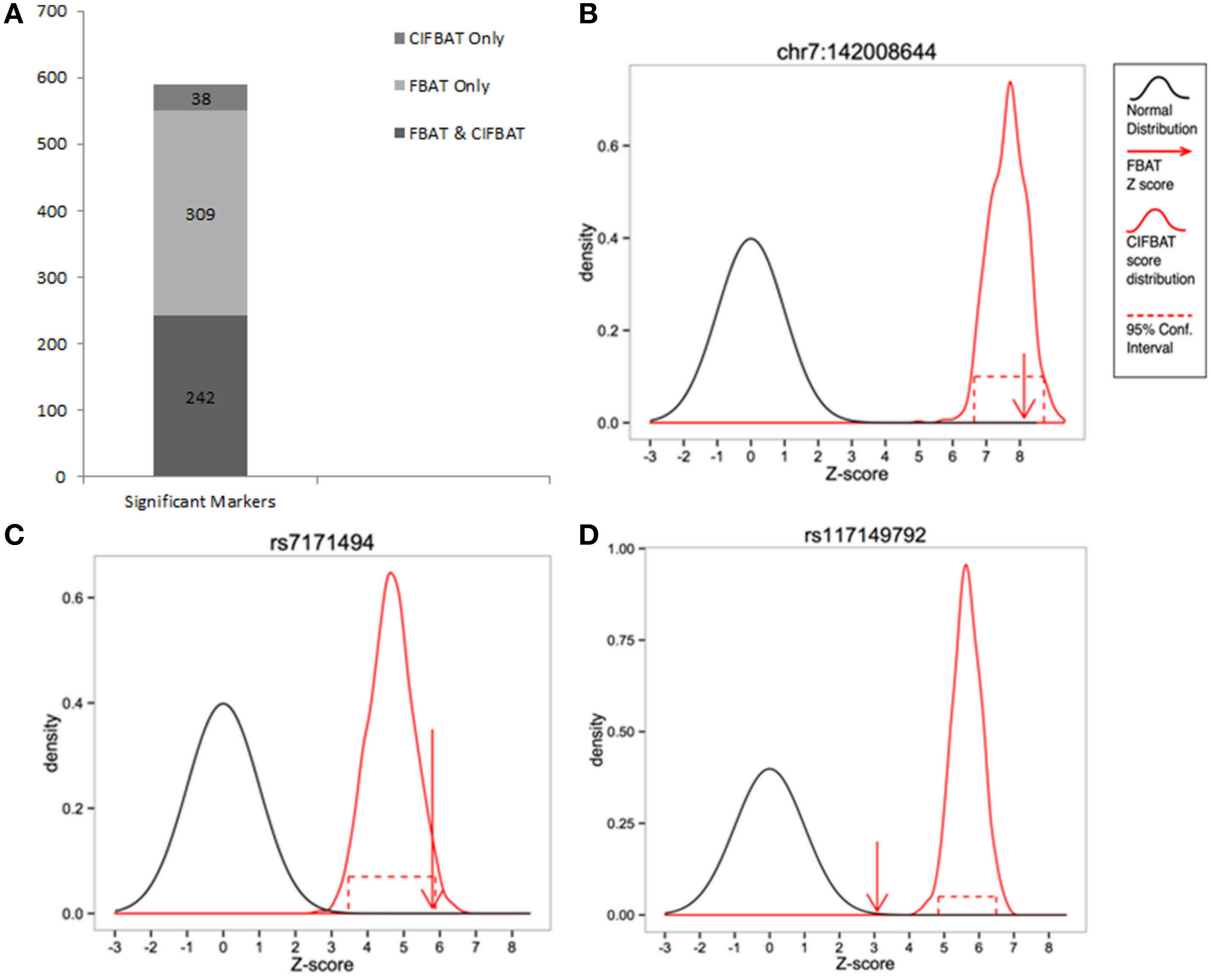
**Analysis of familial whole genome sequencing data for uterine anomalies. (A)** Comparing the number of significant results exclusively and jointly under FBAT and CIFBAT. Of the 551 markers significant under FBAT, 242 (~44%) were validated, and 309 (~56%) were negated by CIFBAT after including incomplete trios in the test. Thirty nine additional markers were identified as significant exclusively by CIFBAT. **(B)** An example of a marker which was significant under FBAT and further validated by CIFBAT. **(C)** An example of a marker which was significant under FBAT, but was not validated by CIFBAT upon inclusion of incomplete trios in the test. **(D)** An example of a marker which was exclusively significant under CIFBAT.

**Figure 5:**
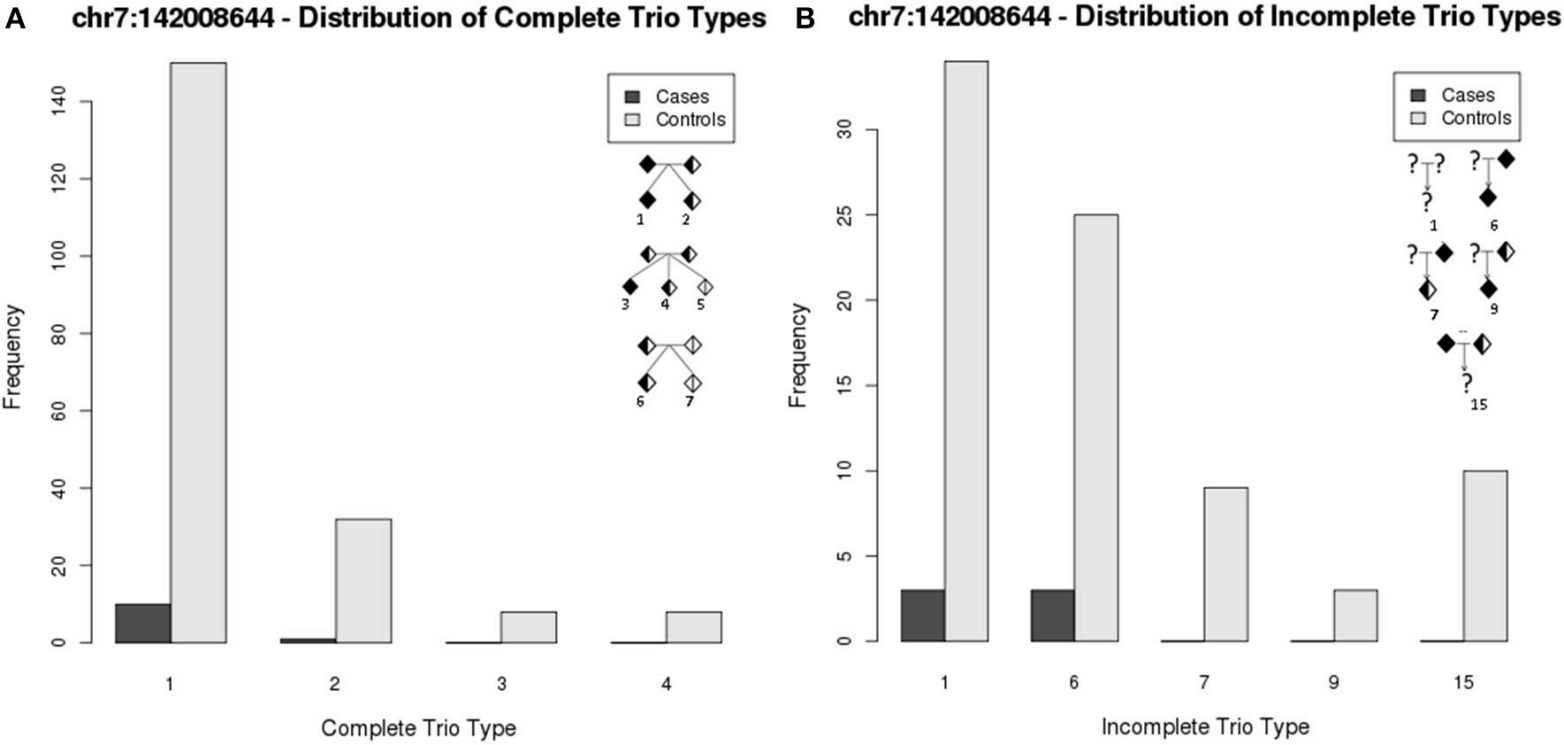
**Distribution of trio types within cases and controls for chr7:142008644. (A)** Complete trio types—Trio type numbers mentioned in the legend correspond to those in the Figure S1. **(B)** Incomplete trio types - Trio type numbers mentioned in the legend correspond to those in the Figure S2. Only trio types that had non-zero counts are shown here.

We compared the counts of significant markers exclusively and jointly identified by FBAT and CIFBAT. Figure 4A shows that out of the 551 markers that were significant under FBAT, 242 (~44%) were also significant under CIFBAT when incomplete trios were included in the test. Validation under CIFBAT indicates the robustness of these results against missing data and serves as a way to select features with higher precision for downstream analyses. All but one of the 242 CIFBAT markers were either intergenic or were positioned in non-coding regions of their respective genes. Figure 4B compares the FBAT statistic and CIFBAT quantile interval for chr7:142008644 (GRCh37), which lies in the second exon of gene *TCRBV9S1A1T*. This marker was significant under FBAT with *p*-value less than machine epsilon (2.22e-16), as well as under CIFBAT with a 95% QI [<2.22e-16, 3.23e-10]. Figure 5 illustrates distribution of complete and incomplete trio types between case and control trios for this marker. A complete list of all the markers significant under both FBAT and CIFBAT can be found in Table S13.

The remaining 309 (~56%) markers, significant under FBAT and not under CIFBAT, indicates that these FBAT results might have been affected by missing data. In such cases, CIFBAT is useful in identifying potential false positives and refining the feature set for further downstream analyses. Example of one such marker is chr15:29443416 (GRCh37; rs7171494) which is an intronic variant in gene *FAM189A1*. Figure 4C compares the FBAT statistic and the CIFBAT QI for this marker, and Figure S10 illustrates the underlying distribution of complete and incomplete trio types between case and control trios. A complete list of all the markers that were significant under FBAT, but could not be validated by CIFBAT can be found in Table S14.

Additionally, there were 38 markers that were exclusively significant under CIFBAT (Table S15). While it is not possible to make conclusive remarks about this set of markers without further independent validation, these could either be categorized as markers where CIFBAT had improved statistical power due to increased sample size or as false positive results. An example marker from this set is chr11:55861880 (rs117149792) which is an upstream gene variant for gene *OR8I2*. Figure 4D compares the FBAT statistic and the CIFBAT QI for this marker, and Figure S11 illustrates the underlying distribution of complete and incomplete trio types between case and control trios.

Overall, CIFBAT can be a useful test to evaluate robustness of FBAT statistic against missing data. Markers that were significant under FBAT and further validated by CIFBAT after inclusion of incomplete families provide a refined set of candidate markers for downstream analysis. In contrast to refining the set of candidate markers by ~50%, CIFBAT exclusively identified only a small fraction (6.8%) of significant markers collectively identified (589) by FBAT and CIFBAT. These can be potentially interesting markers that warrant further investigation. This feature of CIFBAT is particularly useful in genome wide association studies where the initial set of markers to be evaluated for candidacy is invariably very large. CIFBAT can be used in these studies to refine the set of markers for downstream analyses. Moreover, often the underlying genetic model or the pattern of missing data is unknown; in such cases, CIFBAT can be a great advantage in evaluating the effects of missing data without any assumptions about the underlying model.

### Type 1 diabetes data

Table 5 lists results from the analysis of Type 1 Diabetes data for 351 markers within 22 candidate genes. The *p*-values from FBAT were corrected for multiple testing using Benjamini-Hochberg method (Benjamini and Hochberg, 1995). A cut-off of 10% false discovery rate (FDR) was used to define significance. For FBAT, the *p*-value cut-off at 10% FDR was 1.10e-02, which was also used to indicate significant QIs from CIFBAT.

**Table 5:** **Detailed results from analysis of candidate markers for Type I Diabetes**.

**Marker**	**SNP ID**	**FBAT *p*-value**	**CIFBAT 95% Quantile interval (*p*-value)**	**Missing data (%)**	**MAF (%)**
**INS**					
11:2137971	rs3842748	4.44E-16	(<2.22E-16, < 2.22E-16)^*^	22.95	10.95
11:2130023	rs7924316	2.32E-07	(2.18E-09, 9.33E-15)^*^	13.25	49.12
11:2157914	rs11564709	2.21E-07	(<2.22E-16, < 2.22E-16)^*^	13.04	5.37
11:2147527	rs6356	9.00E-07	(3.14E-06, 7.01E-03)	13.27	46.16
11:2151386	rs7119275	1.32E-06	(<2.22E-16, 7.99E-15)^*^	13.42	25.31
11:2126719	rs1004446	4.11E-06	(<2.22E-16, 4.44E-15)^*^	13.24	43.86
11:2124119	rs1003483	4.65E-06	(1.51E-04, 2.29E-08)^*^	13.44	43.17
11:2152413	rs10840495	6.01E-06	(<2.22E-16, 4.93E-14)^*^	13.08	25.46
11:2119686	rs4244808	7.62E-06	(1.36E-07, 8.90E-04)^*^	15.69	41.53
11:2156905	rs11564710	2.81E-04	(1.22E-12, 8.48E-08)^*^	13.09	30.37
11:2154012	rs4929966	6.55E-04	(<2.22E-16, < 2.22E-16)^*^	13.44	17.59
11:2150966	rs10840491	2.85E-03	(1.11E-14, 1.26E-08)^*^	13.13	12.26
**PTPN22**					
1:114089610	rs2476601	2.40E-14	(0.65, 1.53E-02)	13.13	12.17
1:114141503	rs2358994	1.76E-08	(0.85, 4.48E-02)	13.09	17.70
1:114127410	rs2488457	1.16E-07	(0.21, 0.48)	13.04	21.43
1:114132370	rs12566340	1.02E-07	(0.16, 0.66)	13.06	23.20
1:114132504	rs7529353	2.24E-07	(0.21, 0.56)	13.07	23.40
1:114086477	rs1217395	4.98E-07	(0.20, 0.33)	16.89	24.80
1:114138866	rs7524200	5.81E-07	(1.72E-04, 4.16E-02)	13.10	32.80
1:114078476	rs3789607	1.60E-05	(<2.22E-16, 4.44E-15)^*^	13.98	39.95
1:114142398	rs1539438	1.80E-05	(<2.22E-16, 5.60E-13)^*^	13.63	23.91
1:114129479	rs1235005	2.47E-05	(2.04E-02, 3.28E-05)	13.22	38.32
1:114131802	rs1217384	4.29E-05	(1.35E-14, < 2.22E-16)^*^	13.07	22.29
1:114145701	rs1217394	4.55E-05	(<2.22E-16, 3.47E-13)^*^	13.07	24.08
1:114129885	rs6665194	6.89E-05	(2.69E-02, 3.57E-05)	13.49	38.31
1:114063748	rs6537798	7.51E-05	(1.35E-02, 2.39E-05)	13.28	39.43
1:114113273	rs1217418	9.21E-05	(1.58E-05, 1.68E-02)	13.09	39.50
1:114081776	rs2476600	1.09E-04	(1.61E-02, 1.94E-05)	13.09	39.40
1:114056125	rs1217379	1.73E-04	(1.83E-02, 3.20E-05)	13.69	39.37
**CTLA4**					
2:204567056	rs231727	1.62E-03	(0.24, 0.48)	13.32	48.15
2:204566672	rs1427676	3.02E-03	(0.31, 0.37)	13.32	29.98
**IL4R, IL2RA, IL12B**					
16:27281465	rs1805012	5.62E-07	(<2.22E-16, < 2.22E-16)^*^	14.57	0.37
10:6163501	rs12251307	8.15E-04	(<2.22E-16, < 2.22E-16)^*^	13.20	7.81
10:6139051	rs2104286	3.97E-03	(<2.22E-16, 2.22E-16)^*^	13.20	18.71
5:158700244	rs17056704	4.62E-03	(2.10E-07, 8.96E-13)^*^	13.32	23.70
**IFIH1**					
2:162949558	rs1990760	4.32E-03	(1.31E-06, 1.75E-11)^*^	13.32	29.97

FBAT results were corrected for multiple testing using Benjamini-Hochberg method with a 10% false discovery rate cut-off. The corresponding p-value cut-off of 1.10e-02 was also used to indicate significant QI from CIFBAT. Out of the 36 markers significant under FBAT, 20, indicated by an ^*^ following the quantile interval, were validated under CIFBAT showing significant lower and upper bounds of the quantile interval.

**Figure 6:**
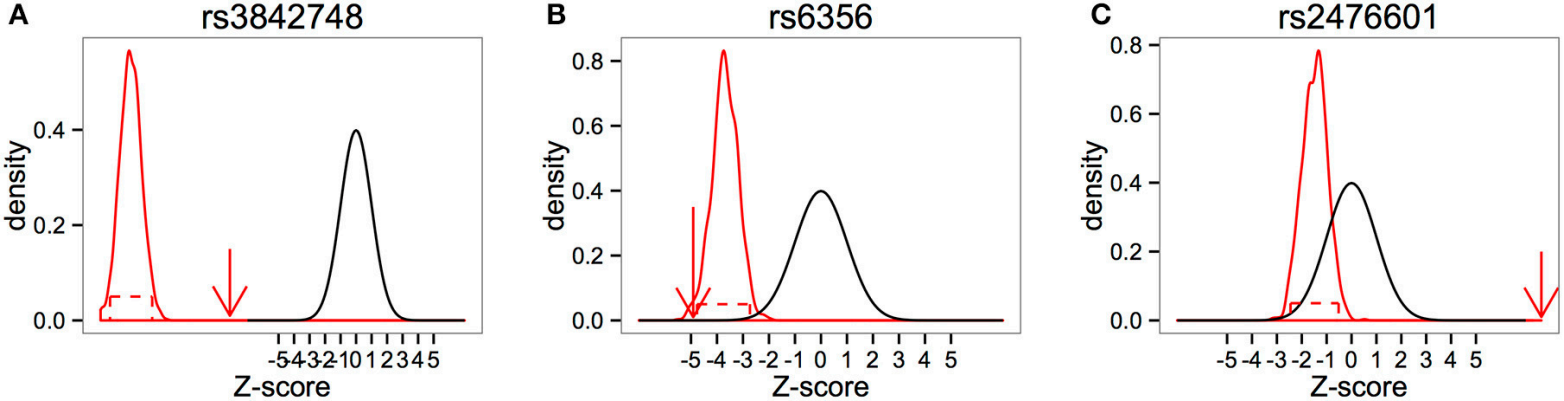
**Analysis of candidate set of markers for Type I Diabetes. (A)** Example of a marker in the INS gene that was significant under FBAT, and further validated by CIFBAT upon inclusion of incomplete trios in the test. **(B)** Example of a marker in the INS gene that could not be validated by CIFBAT. **(C)** Example of a marker in gene PTPN22 which was significant under FBAT, but could not be validated by CIFBAT.

Twelve (12) markers in the insulin (*INS*) gene were identified as significant by FBAT based on complete trios. Of these, 11 markers also showed significant QIs under CIFBAT when incomplete trios were included in the test (example shown in Figure 6A). For *rs6356*, inclusion of incomplete trios caused the distribution of z-scores to overlap with the null distribution making the lower bound of the 95% CI insignificant as shown in Figure 6B.

Seventeen (17) markers in the *PTPN22* gene were identified as significant by FBAT. Of these, 4 were further validated by CIFBAT after inclusion of incomplete trios, but the remaining 13 were not significant anymore (example shown in Figure 6C).

Two (2) markers in the CTLA4 gene were significant under FBAT, but inclusion of incomplete trios under CIFBAT produced insignificant QIs for both the markers.

Four (4) markers in the interleukin (IL4R, IL2RA, IL12B) family of genes, and 1 marker in the *IFIH1* gene were significant under FBAT and were further validated by CIFBAT after inclusion of incomplete trios in the test.

Overall, CIFBAT provided further validation of 20 (55.55%) out of 36 markers that were significant based on FBAT, indicating that these results were not biased by missing data. The remaining 16 markers were not significant anymore when incomplete trios were included under CIFBAT, suggesting that the FBAT test might have been biased due to missing data.

## Discussion

Missing data in genetic association studies pose several challenges. They result in loss of statistical power if samples with missing data are excluded from the study. On the other hand, if missing data is imputed without consideration for the underlying data model, it might lead to often undetectable biases in the results. Here, we have implemented CIFBAT to detect potential bias in FBAT due to the presence of missing data and to identify significant genomic markers with higher precision. Unlike likelihood based methods that use sufficient statistics to impute missing genotypes, CIFBAT considers all valid completions of an incomplete trio equally likely and computes QIs of the FBAT statistic over many randomized iterations. In doing so, CIFBAT does not assume a homogeneous population and retains robustness to population stratification which is a crucial feature of family based association tests.

Using simulated data, we have shown that CIFBAT is useful in validating the robustness of FBAT statistic against missing data and identifying candidate markers with higher precision. We have also demonstrated the applicability of CIFBAT in genome wide association studies, and its usefulness in refining sets of candidate markers for more targeted downstream analyses.

CIFBAT uses a memory efficient data format, making it apt for analyzing whole genome sequencing. We also extended TDT to handle dominant and recessive genetic models in addition to the default additive model, and extended robustTDT to handle the X chromosome in addition to autosomal chromosomes. We have provided a comprehensive software package called FamSuite (https://github.com/IlyaLab/FamSuite) that researchers can use to analyze their data and compare results from TDT, robustTDT, FBAT, and CIFBAT.

## Author contributions

VD conceived the study, wrote the software and conducted analysis of familial whole genome sequences, DG conducted simulation study, TK contributed significantly in method development and analysis guidance, RK contributed toward method development, BB provided critical reviews toward strengthening method and analysis, JV, JN, and IS provided supervision, interpretation of the results and contributed to the writing of the manuscript.

### Conflict of interest statement

The authors declare that the research was conducted in the absence of any commercial or financial relationships that could be construed as a potential conflict of interest.

## Supplementary material

Data Sheet 1 **Simulation parameters, list of uterine anomalies, and whole genome sequence filters**.

Figure S1 **Comprehensive list of informative complete trios. (A)** Autosomal chromosomes, **(B)** X chromosome: trios with female offspring, **(C)** X chromosome: trios with male offspring.

Figure S2 **Comprehensive list of admissible incomplete trios. (A)** Autosomal chromosomes, **(B)** X chromosome: trios with female offspring, **(C)** X chromosome: trios with male offspring.

Figure S3 **Changes in the sensitivity of detecting simulated causative markers, depending on the missing data rate and the level of penetrance in the phenotype model**.

Figure S4 **Changes in the positive predictive value (PPV) when detecting simulated causative markers, depending on the missing data rate and the level of penetrance in the phenotype model**.

Figure S5 **F1 scores for detecting simulated causative markers by missingness scenario, at different missingness rates and FDR thresholds**. The scenarios indicate where missing data was concentrated.

Figure S6 **Precision (TP/TP+FP) for detecting simulated causative markers versus missing data rates**.

Figure S7 **Recall (TP/TP+FN) for detecting simulated causative markers versus missing data rates**.

Figure S8 **Precision (TP/TP+FP) for detecting simulated causative markers by missingness scenario, at different missingness rates and FDR thresholds**. The scenarios indicate where missing data was concentrated.

Figure S9 **Recall (TP/TP+FN) for detecting simulated causative markers by missingness scenario, at different missingness rates and FDR thresholds**. The scenarios indicate where missing data was concentrated.

Figure S10 **Distribution of trio types within cases and controls for chr15:29443416. (A)** Complete trio types—Trio type numbers mentioned in the legend correspond to those in the Figure S1. **(B)** Incomplete trio types—Trio type numbers mentioned in the legend correspond to those in the Figure S2. Only trio types that had non-zero counts are shown here.

Figure S11 **Distribution of trio types within cases and controls for chr11:55861880. (A)** Complete trio types—Trio type numbers mentioned in the legend correspond to those in the Figure S1. **(B)** Incomplete trio types—Trio type numbers mentioned in the legend correspond to those in the Figure S2. Only trio types that had non-zero counts are shown here.

Figure S12 **Z score distributions after randomizing phenotypes**. The density plot **(A)** shows the distribution of Z scores produced when the null (no association) is true. The density plots in **(B)** show the effect missing data rates have on quantile interval widths (wider with more missing data).

Table S1 **Informative trios for autosomal chromosomes and corresponding FBAT statistics for dominant and recessive models**.

Table S2 **Informative trios with female offspring and corresponding FBAT statistics for the X chromosome under dominant and recessive models**.

Table S3 **Admissible incomplete trio types for autosomal chromosomes and the corresponding FBAT statistics X − E[X] and Vartiance(X) for all possible completions of the incomplete trios**.

Table S4 **Admissible incomplete trio types with male offspring and the corresponding FBAT statistics X − E[X] and Vartiance(X) for all possible completions of the incomplete trios for the X chromosome**. Since the father and offspring are haploid and the mother is always heterozygous for these trio types to be informative, all the three genetic models have equivalent risk factors.

Table S5 **Admissible incomplete trio types with female offspring and the corresponding FBAT statistics X − E[X] and Variance (X) for all possible completions of the incomplete trios for the X chromosome**.

Table S6 **Autosomal chromosomes: Transmission counts for alleles *b* and *c* for informative trios for additive, dominant, and recessive models**. Trio types 1 and 2 are not informative under recessive model, and trio types 6 and 7 are not informative under dominant model.

Table S7 **X chromosome: Transmission counts for alleles *b* and *c* for informative trios with male offspring**. Haploid fathers and offspring as well as heterozygous mothers have equivalent risk factors under additive, dominant, and recessive models, hence transmission counts for alleles are also equivalent for the three models.

Table S8 **X chromosome: Transmission counts for alleles *b* and *c* for informative trios with female offspring**.

Table S9 **Admissible incomplete trios for autosomal chromosomes and transmission counts of the two alleles *b* and *c* for all possible completions of the incomplete trios under additive, dominant, and recessive models**.

Table S10 **Admissible incomplete trios with male offspring for the X chromosome and transmission counts of the two alleles *b* and *c* for all possible completions of the incomplete trios**. All the three genetic models have equivalent risk factors because the father and offspring are haploid and the mother is heterozygous for all informative trios.

Table S11 **Admissible incomplete trios with female offspring for the X chromosome and transmission counts of the two alleles *b* and *c* for all possible completions of the incomplete trios**.
