# Supplementary material for: Using Incomplete Trios to Boost Confidence in Family Based Association Studies
Source: Front Genet. 2016 Mar 18;7:34. doi: 10.3389/fgene.2016.00034 (PMC4796035; doi:10.3389/fgene.2016.00034)
Supplement: Data Sheet 1 — Simulation parameters, list of uterine anomalies, and whole genome sequence filters. [file DataSheet1.DOCX]

## Genetic Simulation:

The model is defined as such.

For each marker, by population, the allele frequency follows the gamma distribution with shape=2, scale=2, and scaled by 1/35 to give probabilities between 0.0 and 0.5.

This creates two populations with both similar and diverging allele frequencies.

Then after generating the pedigrees, the probability for disease is given by:

Log(π_ij_) = α_ij_ + Σ β_m_X(G_ijm_)

Where α_ij_ ~ MN(μ, Σ)

f_2_ ~ N(μ, σ)

f_0_ ~ N(μ, σ)

f_1_ = (f_2_ + f_0_)/2

μ = log(f_0_) – σ^2^/2

σ^2^ = (1/2)log(λ_s_/ λ_g_**)**

β_m_ = vector { log(f_2_)/log(f_0_), log(f_1_)/log(f_0_), 0.0 }

X(G_ijm_) = vector indicating number minor alleles (i.e. for one minor allele: {0,1,0})

Parameters used:

Number Populations = 2

Population 2 / Population 1 =~ 0.33

Number Pedigrees = 4000

Number Children = uniform random (1,2)

Cases / Controls =~ 1.0

Number of markers = 300

Number causative = 3

f_2_ ~ N(0.1, 0.01)

f_0_ ~ N(0.001, 0.001)

λ_s_ = 3

λ_g_ = 2

CIFBAT trials = 200

**Uterine Anomalies**

 Endometriosis

 Recurrent Antepartum Hemorrhage

 bicornuate uterus

 thin lower uterine segment

 uterine didelphys

 uterine leiomyomata

 bulging lower uterine segment

 uterine scar

 uterine fibroid

 large left uterine fibroid

 uterine atony

 bicornuate uterus

 didelphic uterus

**Filters applied to WGS family data for maternal uterine anomalies**

- **Call rate >90%**
- **Bi-allelic markers**
- **MAF > 1%**
- **Commonly Mutated Segments**

In an independent study, we analyzed a set of 510 independent genomes (produced using Complete Genomics technology and separate from the genomes in the cohort used in the current study) to identify regions of the genome frequently observed as mutated. We collected 42,524,348 SNVs from the independent genomes and tested the functional effect of each variant using Functional Annotation of Variants (FAVA), a pre-computed genome-wide database of predicted functional effects of specific point mutations and of annotated functional ranges. We retained observed variants predicted to cause coding disruptions in a comprehensive set of transcript annotations and predictions obtained from the UCSC database, including: knownGene, ensGene, acembly, ccdsGene, refGene, exoniphy, sibGene, vegaGene, hinv70Coding, hinv70NonCoding, mgcGenes and xenoRefGene. This analysis yielded a set of 513,985 variants predicted to cause nonsense, missense, termination site loss or splice disruption changes to at least one transcript. We then identified clusters of such disruptions by imposing a maximal distance of 500 bp between consecutive sites in a cluster, and requiring clusters to be at least 10 bp long. We defined the number of ‘disruption events’ in a cluster as the sum of the number of individuals presenting each SNV in the cluster, and computed an ad-hoc cluster density score as the number of disruption events divided by the cluster length plus the number of genomes sampled. We retained 30,751 clusters with score at least 0.1, spanning a total of 14.39 Mb; 2,434 clusters (spanning a total of 0.95 Mb) have score at least 1.0.

- **Extreme Heterozygous Markers**

We analyzed the phase1 release of the 1000 Genomes data to identify sites displaying excess heterozygosity. For each single-nucleotide variant (SNV), we computed the number of homozygous reference, heterozygous, and homozygous variant individuals, stratified into the 14 populations reported in the data set (ASW, CEU, CHB, CHS, CLM, FIN, GBR, IBS, JPT, LWK, MXL, PUR, TSI and YRI). From these individual counts, we computed for each SNV in each population the variant allele frequency and thus the expected number of heterozygous individuals. We then integrated these values across all populations, and computed the total observed and total expected number of heterozygous individuals. We selected as excess heterozygous sites SNVs with an observed/expected ratio at or above 1.9, and for which at least 50 heterozygous individuals were expected after combining all populations. This analysis identified a set of 20488 SNVs for which almost all individuals are observed to be heterozygous.
