# Supplementary figures and images for: Using Incomplete Trios to Boost Confidence in Family Based Association Studies
Source: Front Genet. 2016 Mar 18;7:34. doi: 10.3389/fgene.2016.00034 (PMC4796035; doi:10.3389/fgene.2016.00034)

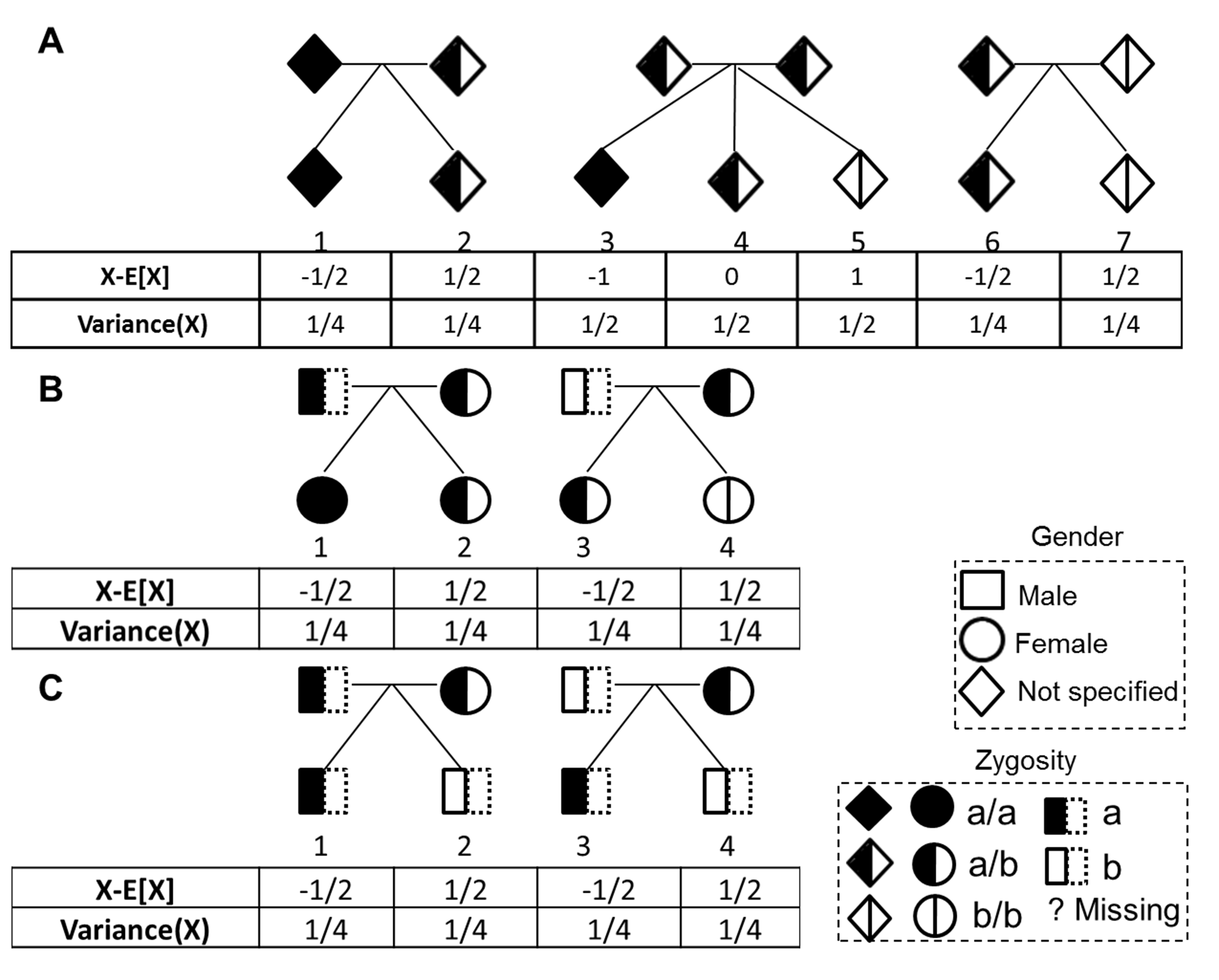

Supplement: Figure S1 — Comprehensive list of informative complete trios. (A) Autosomal chromosomes, (B) X chromosome: trios with female offspring, (C) X chromosome: trios with male offspring. [file Image1.TIF]

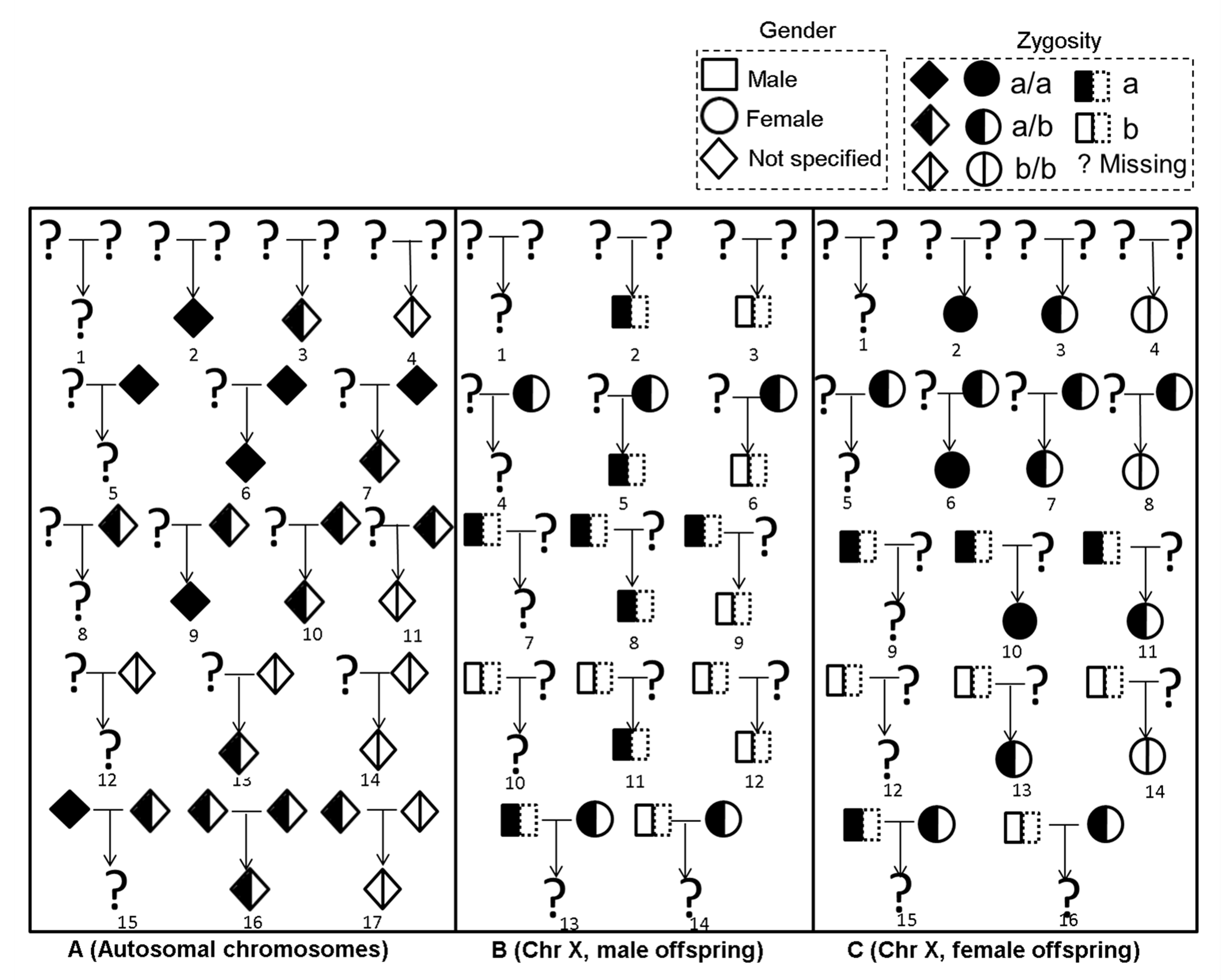

Supplement: Figure S2 — Comprehensive list of admissible incomplete trios. (A) Autosomal chromosomes, (B) X chromosome: trios with female offspring, (C) X chromosome: trios with male offspring. [file Image2.TIF]

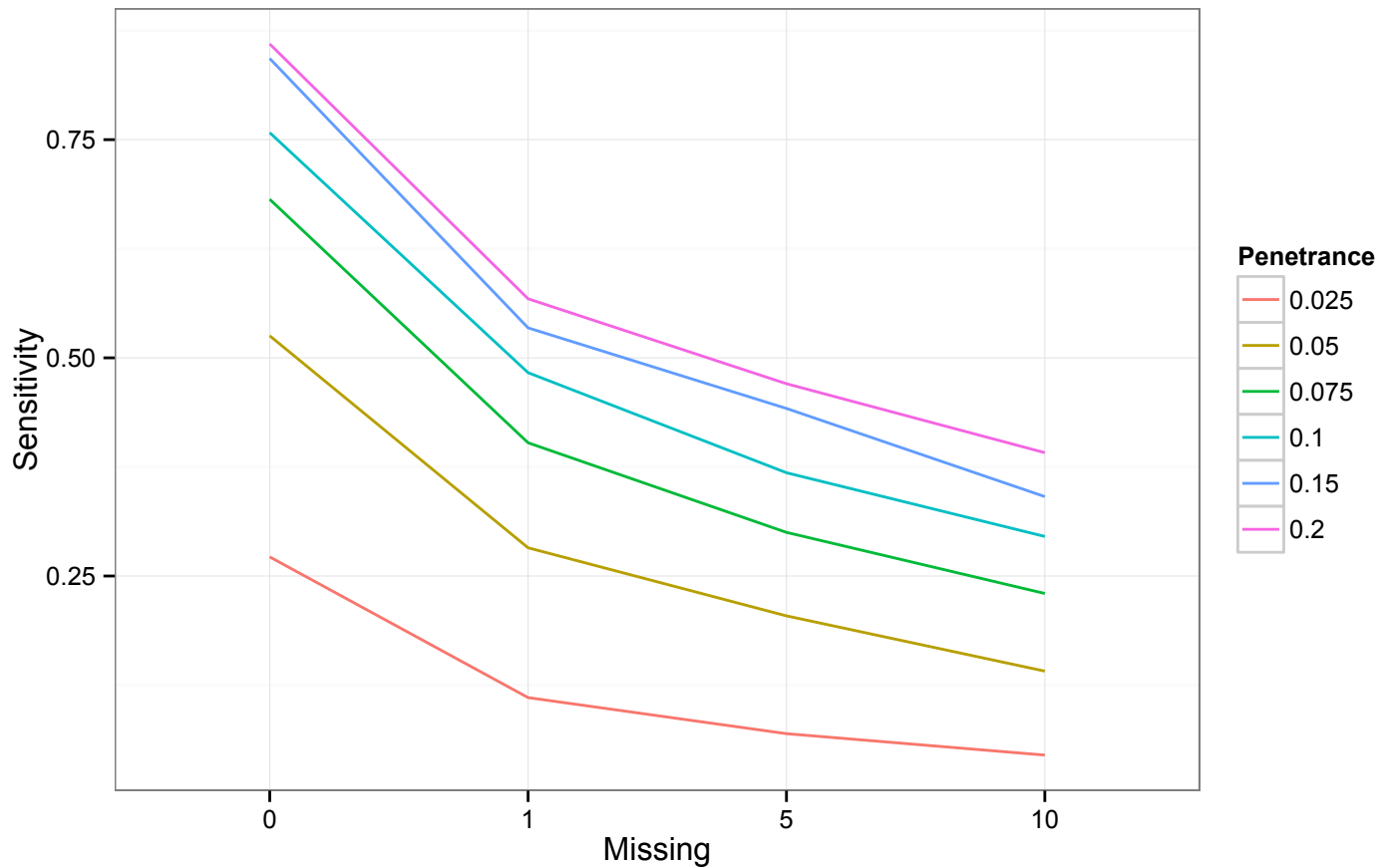

Supplement: Figure S3 — Changes in the sensitivity of detecting simulated causative markers, depending on the missing data rate and the level of penetrance in the phenotype model. [file Image3.PDF]

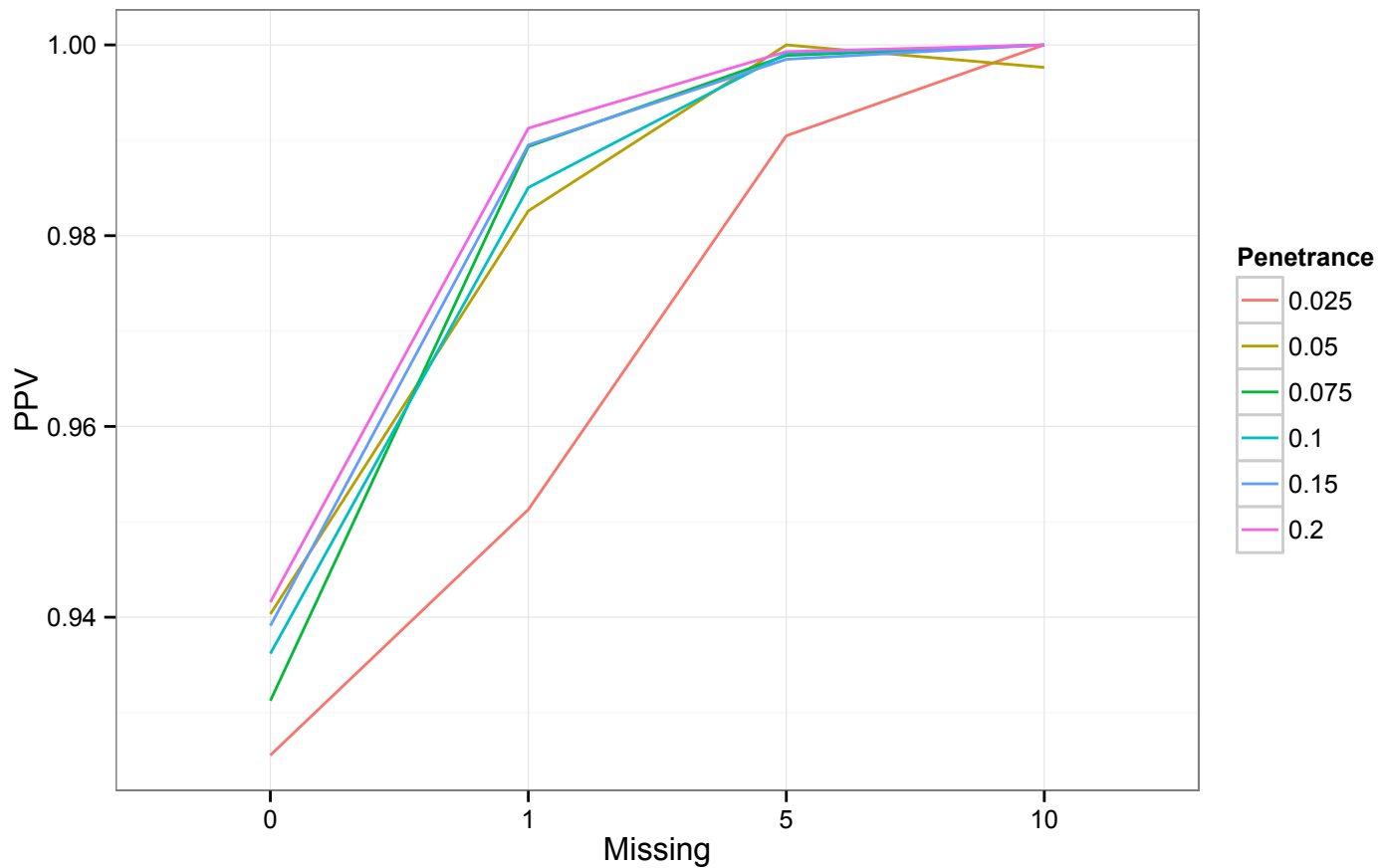

Supplement: Figure S4 — Changes in the positive predictive value (PPV) when detecting simulated causative markers, depending on the missing data rate and the level of penetrance in the phenotype model. [file Image4.PDF]

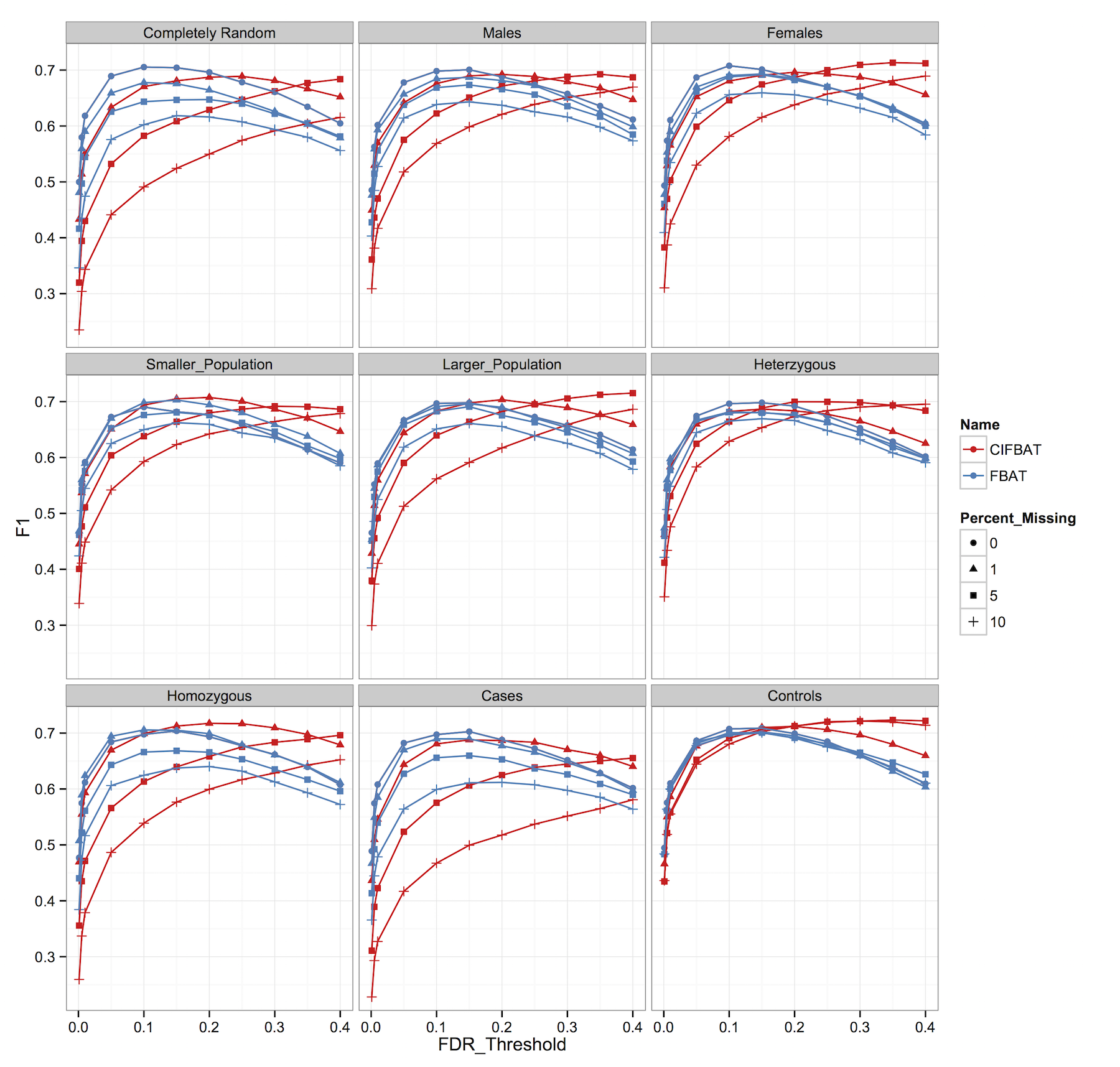

Supplement: Figure S5 — F1 scores for detecting simulated causative markers by missingness scenario, at different missingness rates and FDR thresholds. The scenarios indicate where missing data was concentrated. [file Image5.TIFF]

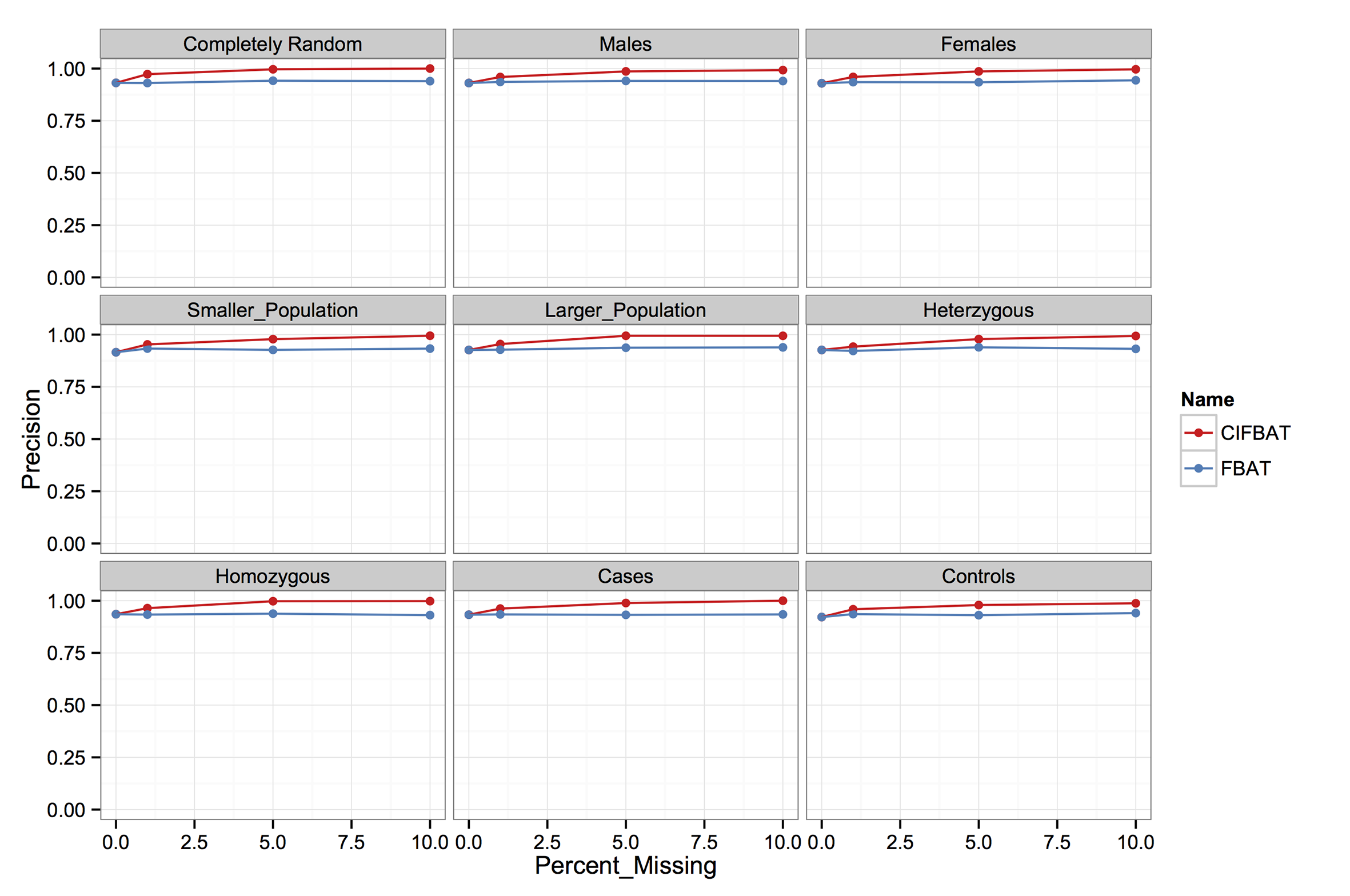

Supplement: Figure S6 — Precision (TP/TP+FP) for detecting simulated causative markers versus missing data rates. [file Image6.TIFF]

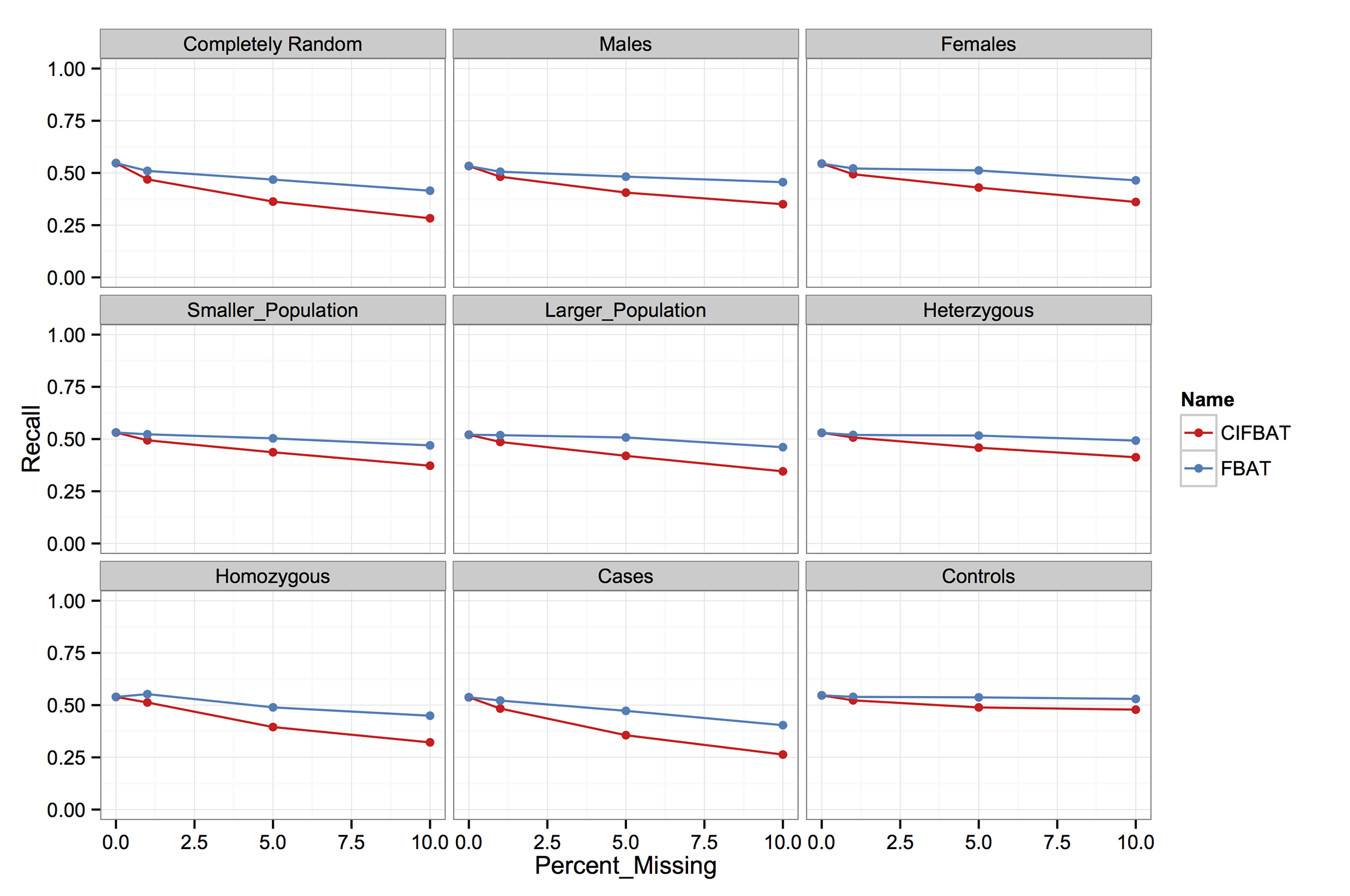

Supplement: Figure S7 — Recall (TP/TP+FN) for detecting simulated causative markers versus missing data rates. [file Image7.TIFF]

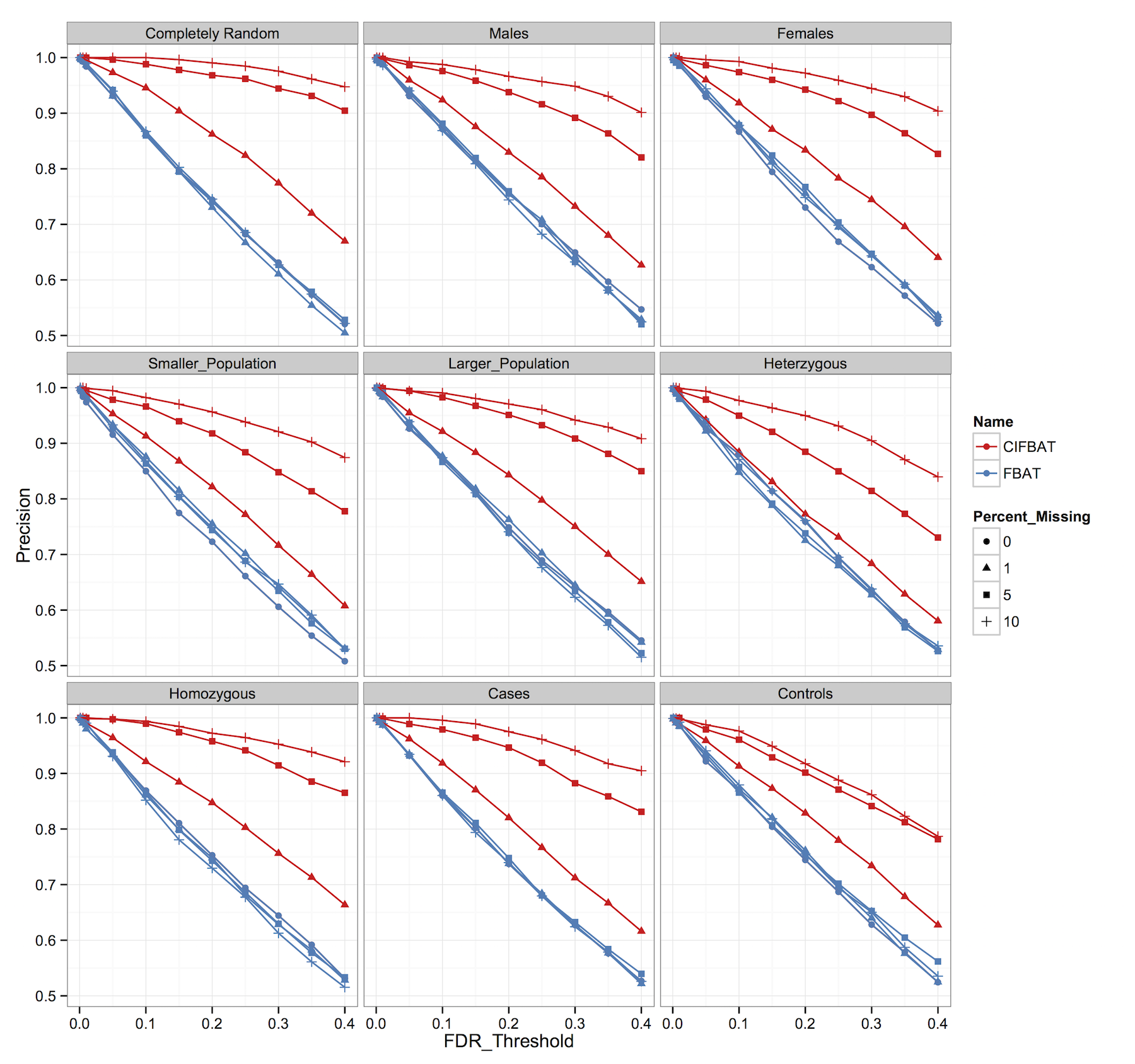

Supplement: Figure S8 — Precision (TP/TP+FP) for detecting simulated causative markers by missingness scenario, at different missingness rates and FDR thresholds. The scenarios indicate where missing data was concentrated. [file Image8.TIFF]

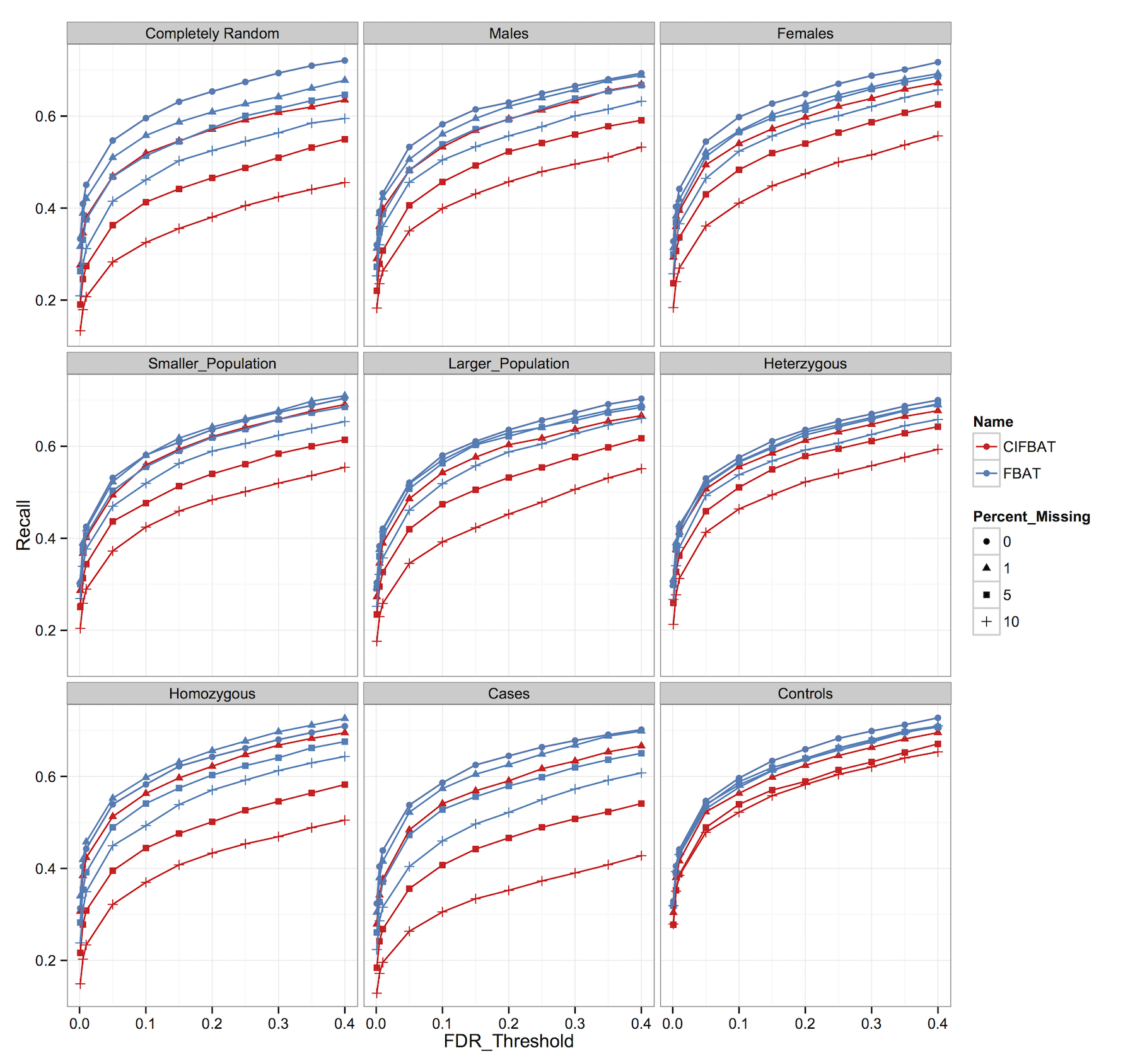

Supplement: Figure S9 — Recall (TP/TP+FN) for detecting simulated causative markers by missingness scenario, at different missingness rates and FDR thresholds. The scenarios indicate where missing data was concentrated. [file Image9.TIFF]

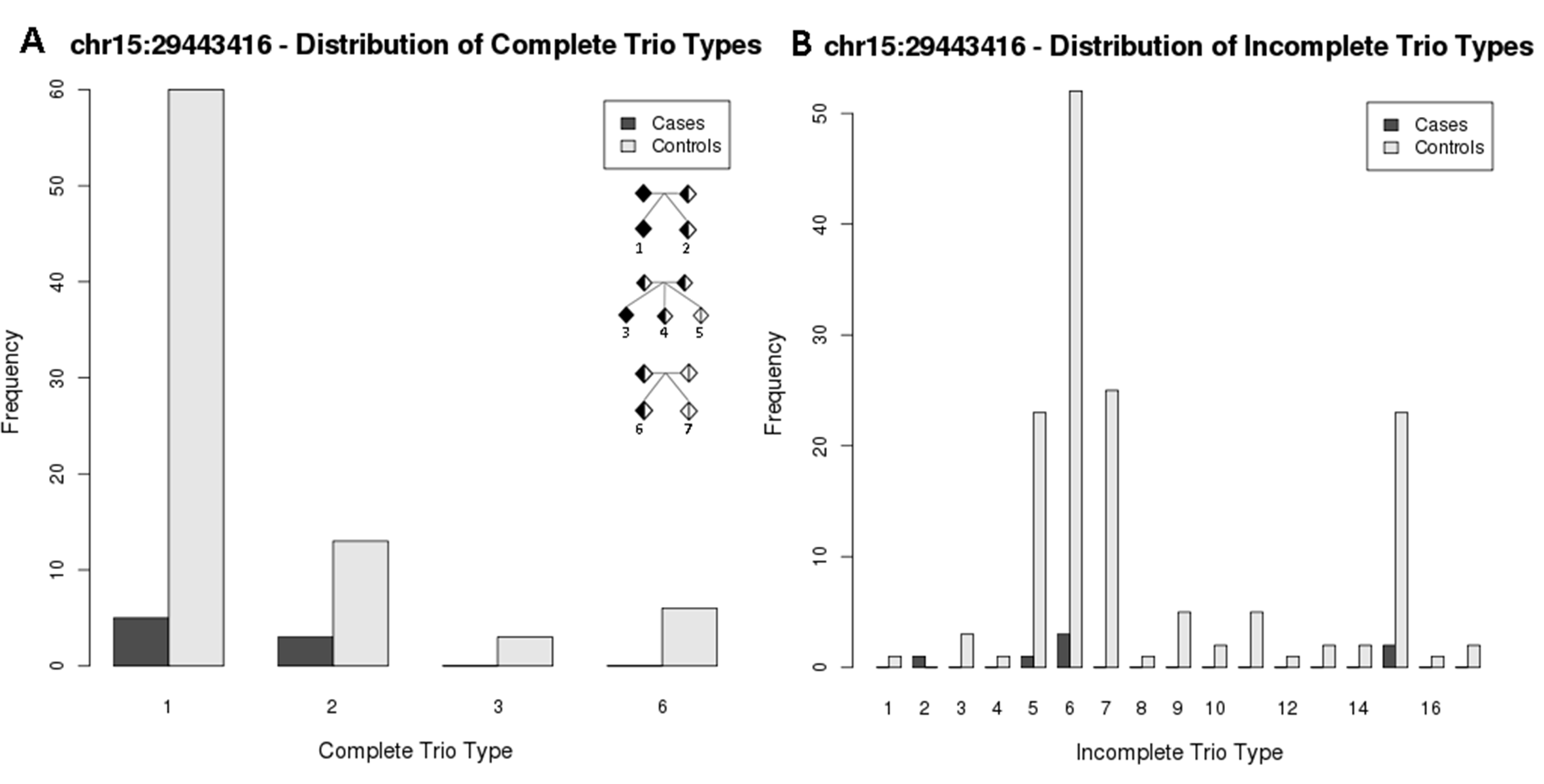

Supplement: Figure S10 — Distribution of trio types within cases and controls for chr15:29443416. (A) Complete trio types—Trio type numbers mentioned in the legend correspond to those in the Figure S1. (B) Incomplete trio types—Trio type numbers mentioned in the legend correspond to those in the Figure S2. Only trio types that had non-zero counts are shown here. [file Image10.tiff]

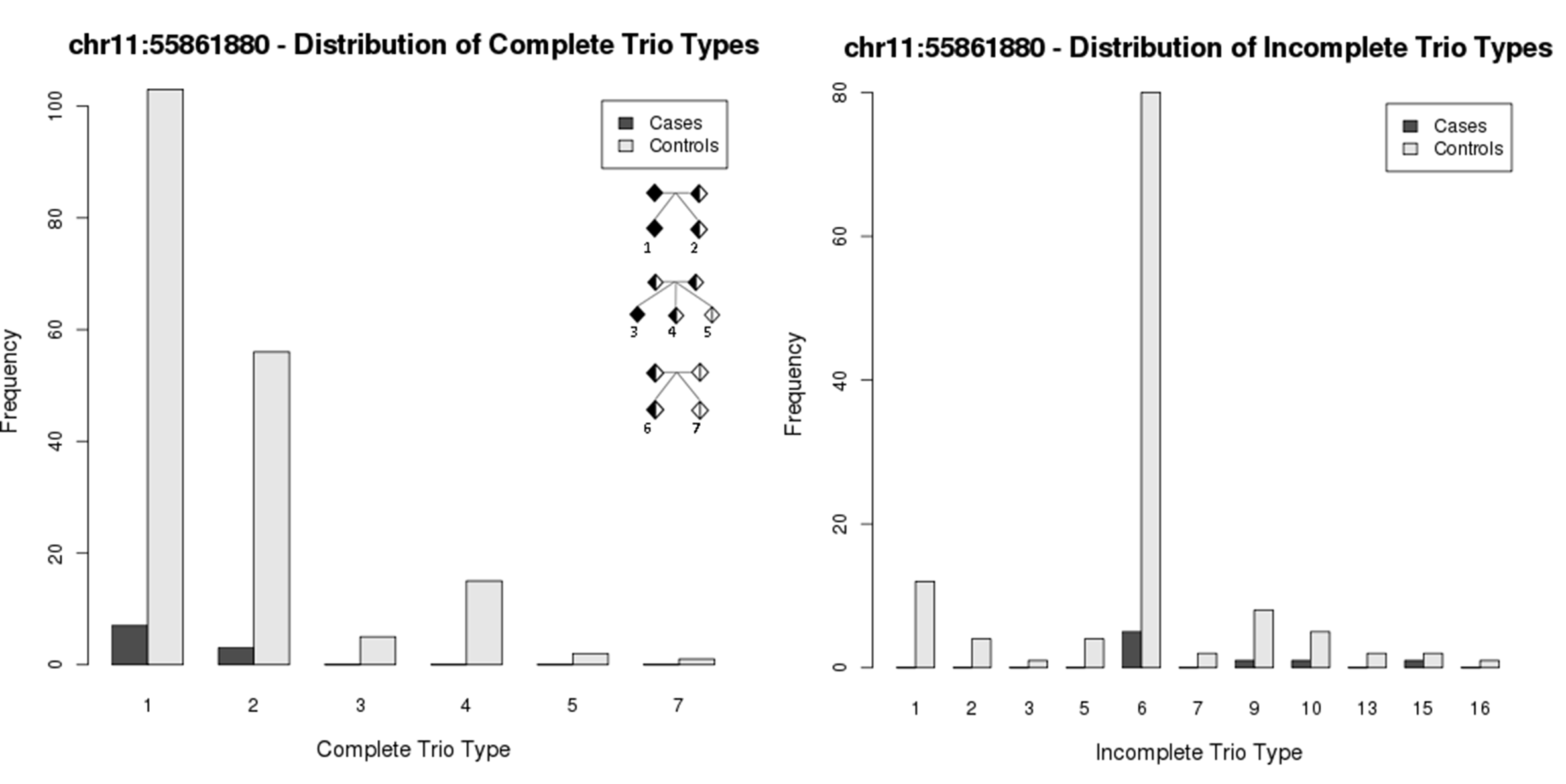

Supplement: Figure S11 — Distribution of trio types within cases and controls for chr11:55861880. (A) Complete trio types—Trio type numbers mentioned in the legend correspond to those in the Figure S1. (B) Incomplete trio types—Trio type numbers mentioned in the legend correspond to those in the Figure S2. Only trio types that had non-zero counts are shown here. [file Image11.TIF]

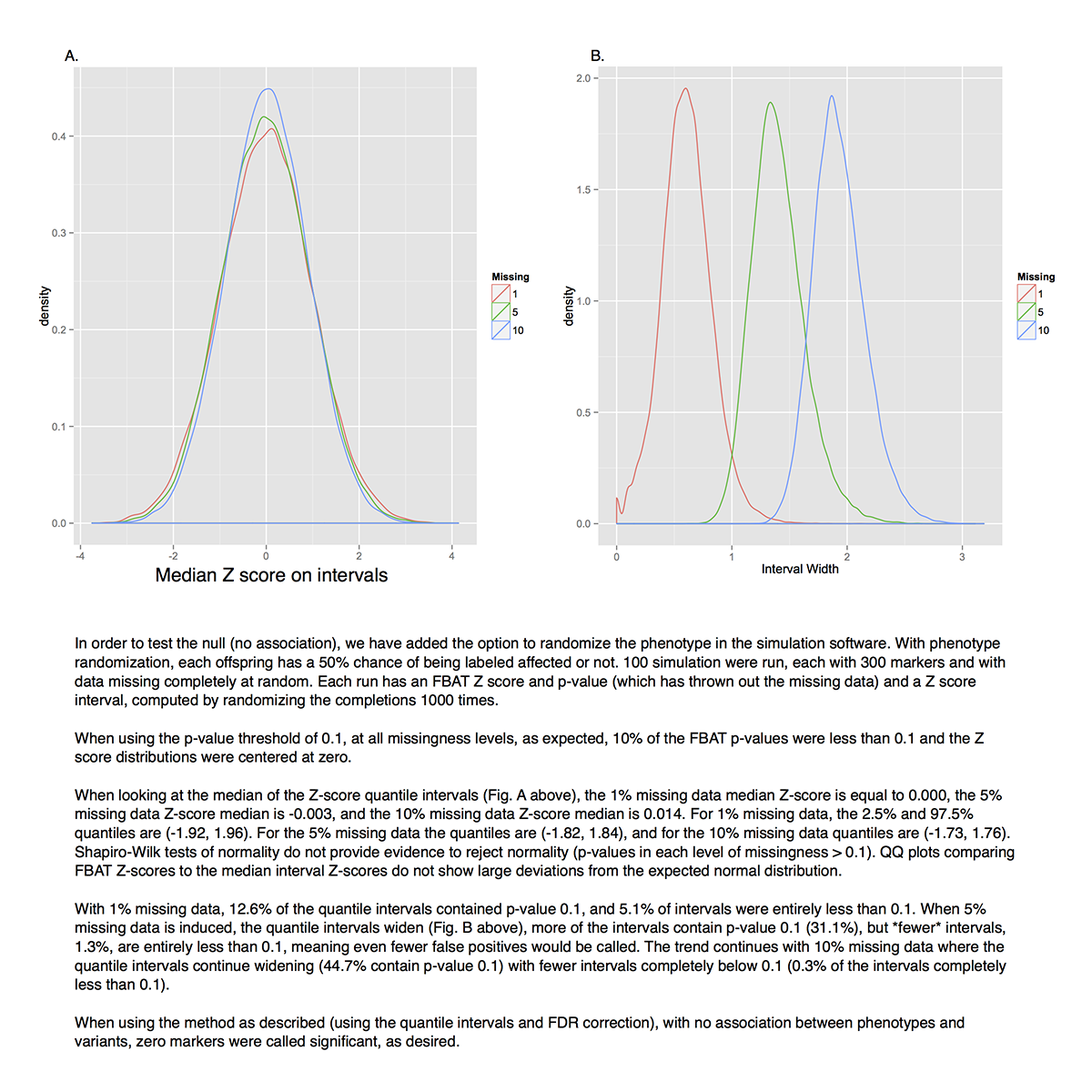

Supplement: Figure S12 — Z score distributions after randomizing phenotypes. The density plot (A) shows the distribution of Z scores produced when the null (no association) is true. The density plots in (B) show the effect missing data rates have on quantile interval widths (wider with more missing data). [file Image12.TIFF]
